# Multi-Omic Analysis in a Metabolic Syndrome Porcine Model Implicates Arachidonic Acid Metabolism Disorder as a Risk Factor for Atherosclerosis

**DOI:** 10.3389/fnut.2022.807118

**Published:** 2022-02-23

**Authors:** Song-Song Xu, Xiu-Ling Zhang, Sha-Sha Liu, Shu-Tang Feng, Guang-Ming Xiang, Chang-Jiang Xu, Zi-Yao Fan, Kui Xu, Nan Wang, Yue Wang, Jing-Jing Che, Zhi-Guo Liu, Yu-Lian Mu, Kui Li

**Affiliations:** ^1^State Key Laboratory of Animal Nutrition and Key Laboratory of Animal Genetics, Breeding and Reproduction of Ministry of Agriculture and Rural Affairs of China, Institute of Animal Sciences, Chinese Academy of Agricultural Sciences, Beijing, China; ^2^Shenzhen Branch, Guangdong Laboratory of Lingnan Modern Agriculture, Genome Analysis Laboratory of the Ministry of Agriculture and Rural Affairs, Agricultural Genomics Institute at Shenzhen, Chinese Academy of Agricultural Sciences, Shenzhen, China; ^3^College of Animal Science and Technology, Nanjing Agricultural University, Nanjing, China; ^4^Animal Husbandry and Veterinary Department, Beijing Vocational College of Agriculture, Beijing, China

**Keywords:** atherothrombosis, metagenome, metatranscriptome, metabolome, transcriptome, inflammatory response

## Abstract

**Background:**

The diet-induced gut microbiota dysbiosis has been suggested as a major risk factor for atherothrombosis, however, the detailed mechanism linking these conditions is yet to be fully understood.

**Methods:**

We established a long-term excessive-energy diet-induced metabolic syndrome (MetS) inbred Wuzhishan minipig model, which is characterized by its genetic stability, small size, and human-like physiology. The metabolic parameters, atherosclerotic lesions, gut microbiome, and host transcriptome were analyzed. Metabolomics profiling revealed a linkage between gut microbiota and atherothrombosis.

**Results:**

We showed that white atheromatous plaque was clearly visible on abdominal aorta in the MetS model. Furthermore, using metagenome and metatranscriptome sequencing, we discovered that the long-term excessive energy intake altered the local intestinal microbiota composition and transcriptional profile, which was most dramatically illustrated by the reduced abundance of SCFAs-producing bacteria including Bacteroides, Lachnospiraceae, and Ruminococcaceae in the MetS model. Liver and abdominal aorta transcriptomes in the MetS model indicate that the diet-induced gut microbiota dysbiosis activated host chronic inflammatory responses and significantly upregulated the expression of genes related to arachidonic acid-dependent signaling pathways. Notably, metabolomics profiling further revealed an intimate linkage between arachidonic acid metabolism and atherothrombosis in the host-gut microbial metabolism axis.

**Conclusions:**

These findings provide new insights into the relationship between atherothrombosis and regulation of gut microbiota via host metabolomes and will be of potential value for the treatment of cardiovascular diseases in MetS.

## Introduction

Recent evidence suggests that excessive energy intake can be a major risk factor for metabolic syndrome (MetS), which a common cause of death worldwide, especially in developed countries. The increasing prevalence of MetS is concomitant with increased incidences of atherothrombosis (1, 2). Atherosclerosis is characterized by the formation of a fibrous plaque or atheroma in the arterial intima, which generally involves medium and large arteries. Several studies demonstrate that chronic inflammation is also a critical factor that promotes the progression of atherosclerosis (3). The over-consumption of excessive energy induces macrophage infiltration and inflammation in the adipose tissue and increases serum lipids and pro-inflammatory cytokines, which further contribute to the inflammation process. More importantly, targeting inflammation has been utilized as a diagnostic marker to treat atherosclerosis in the clinic (4).

Accumulating studies suggest that gut microbiota can directly affect atherosclerosis development via metabolite production of components such as bile acids, coprostanol, short chain fatty acids (SCFAs), and trimethylamine-N-oxide production (5–7). These metabolites are associated with immune dysregulation and exert pro-inflammatory and anti-inflammatory influences. Karlsson et al. (8) revealed that microbial genes that encode peptidoglycan synthesis were enriched in gut metagenomes in atherosclerotic patients. In addition, gut microbiota can lead to atherothrombosis via indirect pathways, including by the modulation of inflammatory cytokines (9).

Currently, the causal relationship between gut microbiota and atherothrombosis remains largely unexplored due to challenges associated with controlling many factors. Firstly, the relationship between excessive energy intake and atherothrombosis prompted studies of inflammation, but most of these studies have been done during relatively short-term dietary intervention periods. Evidence suggests that long-term dietary interventions have a larger impact on host physiology, including gut microbiota and the cardiovascular system, while short-term interventions have temporary effects (10). Secondly, atherosclerotic lesions have been considered as the focus of cardiovascular research, and there is significant difficulty in directly obtaining human specimens for research due to ethical reasons. The development appropriate an animal model with a cardiovascular system significantly similar to the human cardiovascular systems is of vital importance. Studies show that pig and human physiological activities are highly similar with regard to cardiovascular systems, gut microbiome colonization, and metabolic/immune functions (1, 11). Inbred Wuzhishan minipigs, which originated from inbreeding one male and one female Wuzhishan minipig by full-sib mating in 1987, are characterized by its small adult size, genetic stability, and are tolerant to coarse fodder. These pigs provide us with genetically identical test animals to achieve good reproducibility in laboratory experiments (12). Of particular note, the inbred minipigs are typically fed a high-energy diet that can perfectly mimic the progression of human-like diet-induced atherosclerosis (13, 14). Therefore, the inbred model possesses the best balance between human-like physiology and operability.

Gut microbiota are highly structured and specialized in biological functions across different gut segments and selectively harbor characteristic microbes along the longitudinal axis of the gastrointestinal tract (15). Due to limitations associated with collecting small-intestine contents from humans, most of the available gut microbiome studies in humans have used stool samples, which only represent the large-intestinal microbiome. The small-intestinal microbiota is mainly responsible for protein, lipid, carbohydrate, and amino acid metabolism (16). The MetS is recognized as the leading cause of chronic liver disease (e.g., non-alcoholic fatty liver disease), which significantly impacts the synthesis of bile acids (17, 18). Bile acid metabolism can affect gut microbiota composition and inhibit microbial growth in segments of the small-intestine, and this interaction was previously shown to regulate atherothrombosis (19). However, the manner in which the host-gut microbial metabolism axis affects atherothrombosis induced by MetS is largely unknown.

In this study we aimed to investigate the relationship between gut microbiota and atherothrombosis in a MetS porcine model. Herein, our findings elucidated the host-gut microbial metabolism axis mechanism responsible for atherothrombosis in the MetS model. Furthermore, we uncovered the remodeling of gut microbiota that resulted in increased arachidonic acid metabolism, which further led to an inflammatory response in the liver and abdominal aorta. These findings demonstrate that modifications of gut microbiota and alterations of arachidonic acid metabolism have potential as treatments for atherothrombosis.

## Materials and Methods

### Animal Experiments

All experimental protocols were approved by the Animal Care and Use Committee of the Germplasm Resource Center of Chinese Experimental Minipigs. These experiments used a total of 22 male inbred Wuzhishan minipigs obtained from the Chinese Academy of Agricultural Sciences (CAAS), aged 3–4 months and weighing 9.0–11.0 kg. All minipigs were randomly and evenly assigned to two groups: the ND group, in which the minipigs were fed with the normal chow diet according to the NRC nutrient requirements standards recommended for pigs (20); the HED group, in which the pigs were fed with a high-energy diet. The HED formula: 3% cholesterol, 10% fat (lard), and 87% base material. The base material consisted 48% corn, 20% wheat, 15% soybean cake, 12% rice bran, and 5% fish meal. Pigs were housed individually in pens under controlled conditions (temperature 22–25°C, relative humidity 30–70%).

### Serum Biochemistry

Blood samples were collected from jugular veins after overnight fasting. The serum was isolated by centrifuging at 3,500 rpm for 10 min at 4°C. Serum total cholesterol (TC), triglyceride (TG), high-density lipoprotein cholesterol (HDL-C), and low-density lipoprotein cholesterol (LDL-C) levels were determined using the Bayer Advia 1650 Clinical Chemistry System. Additionally, the expression levels of serum cytokines were detected by enzyme-linked immunosorbent assay (ELISA) according to the MILLIPLEX MAP Kit (PCYTMG-23K-13PX) instructions after 64 months of dietary intervention. The stored serum for each individual was thawed for C-reactive protein (CRP) measurement by the ELISA kit (MK2607A, China) according to the manufacturer's instructions. Serum lipopolysaccharides (LPS) concentrations were measured with the ELISA kit (MK9836A, China) following the manufacturer's instructions.

### Pathological Examination

After 64 months of dietary intervention, eight minipigs (HED group: *n* = 4; ND group: *n* = 4) were sacrificed for pathology analysis, microbial metagenome and metatranscriptome, and host transcriptome sequencing analysis. We performed systematic anatomy and examination, with a major focus on liver and abdominal aorta. Hepatocytes from the HED and ND groups were fixed in 4% paraformaldehyde and stained with Oil Red O and TUNEL to examine lipid droplets and cell apoptosis levels (21), respectively. We longitudinally incised the abdominal aortas to observe the extent of atherosclerotic lesions formation. Then, the abdominal aorta was fixed with 4% paraformaldehyde and 2.5% glutaraldehyde and observed by scanning electron microscopy. Furthermore, the abdominal aorta was stained with Oil Red O and Masson's trichrome after fixation with 4% paraformaldehyde to examine intercellular lipid accumulation and foam cells formation, respectively. The ileal tissue was fixed in 4% paraformaldehyde fixative, dehydrated, embedded, and then cut into 3–8 mm-thick sections. For histopathology, the sections were stained by H&E.

### Intestinal Epithelial Barrier Disruption Analysis

The gut section tissues from duodenum, ileum, cecum, colon, and rectum segments were collected from the HED and ND group animals. The mRNA expression levels of tight junction proteins (i.e., Claudin, Occludin, ZO-1, and E-cadherin) were determined by performing qPCR. The PCR primer sequences can be found in Supplementary Table 1. PCR conditions were as follows: 30 s at 95°C, 5 s at 95°C, and 34 s at 60°C (40 cycles). The normalized expression levels were calculated using the 2^−Δ*ΔCt*^ method using QuantStudio^TM^ Design & Analysis Software v1.4.3. The ileal tissues were fixed in 4% paraformaldehyde and processed for paraffin sectioning. The sections were deparaffinized in xylene and hydrated by serial immersion in ethanol and PBS. The sections were incubated with specific primary antibody (1:100, anti-occludin antibody ab216327) overnight at 4°C, washed with PBS, and incubated with fluorophore-conjugated secondary antibody (ZF-0516, Alexa Fluor594-Conjugated AffiniPure Goat Anti-Rabbit IgG) for 1 h at room temperature. The sections were subsequently counterstained with 4,6-diamidino-2-phenylindole (DAPI) and mounted with antifade reagent. The sections were analyzed using a Biorevo BZ-9000 (Keyence) fluorescent microscope.

### Western Blotting

Total protein was extracted from intestinal tissues using the NE-PER Extraction Reagent Kit (Pierce, Waltham, MA, USA) following the manufacturer's instructions. The samples were separated by SDS-PAGE, then transferred to a polyvinylidene fluoride membrane (Millipore, Burlington, MA, USA). The membrane was blocked with skim milk then incubated with primary antibody at 4°C overnight and secondary antibody at room temperature for 1 h. Chemiluminescent signals were captured with a Tanon-520 imager (Tanon, Shanghai, China). Anti-occludin antibody (ab216327, Abcam, MA, USA) was used to detect porcine Occludin, and anti-rabbit IgG HRP-linked antibody (7074S, Cell Signaling Technology, Danvers, MA, USA) was used as the secondary antibody.

### DNA and RNA Extraction, Metagenome, and Metatranscriptome Sequencing

The indicated intestinal (i.e., ileum, cecum, and colon) contents and feces were collected from the HED and ND groups. These specimens were immediately snap-frozen in liquid nitrogen and stored at −80°C for subsequent analyses. Total DNA was extracted based on the repeated bead-beating plus column method (22). The DNA concentration and purity were evaluated using Qubit^®^ dsDNA Assay Kit in a Qubit^®^ 2.0 Flurometer (Life Technologies, CA, USA). Metagenomic DNA libraries were generated using an Illumina Nextera XT kit (Illumina, Inc., San Diego, CA, USA) followed by whole genome sequencing performed on an Illumina HiSeq2500 platform with a 2 × 150-bp, paired-end run. Briefly, the genomic DNA shearing by ultra-sonication was first performed with the Covaris^®^ S2 systems (Covaris Inc., MA, USA). The fragmented DNA was then end-polished, A-tailed, and ligated with an indexed Illumina sequencing adapter. Each metagenomic library was quantified using an Agilent2100 bioanalyzer (Agilent, Böblingen, DE).

Total RNA was extracted using the TRIzol reagent (Invitrogen, Shanghai, China), and treated with RNase-free DNase I (TaKaRa, Shanghai, China) to remove residual genomic DNA. The RNA concentration and purity were evaluated using a Qubit^®^ RNA HS Assay Kit on a Qubit^®^ 2.0 Fluorometer (Life Technologies, Grand Island, NY, USA), and the ribosomal RNA was depleted using an Epicenter Ribo-zero™ rRNA Removal Kit (Epicenter, USA). RNA-Seq libraries were prepared for whole transcriptome sequencing using the NEBNext^®^ Ultra™ Directional RNA Library Prep Kit for Illumina^®^ (NEB, Beverly, USA) according to the manufacturer's protocol, and index codes were added to attribute sequences to each sample. Sequencing of the library preparations for all samples was generated on an Illumina HiSeq2500 platform with a 2 × 150-bp paired-end reads.

### Metagenome and Metatranscriptome Sequence Analysis

Raw reads were cleaned to exclude Illumina adapter sequences, low-quality sequences, and host genomic DNA, and to form a clean data reads. The clean reads were then assembled using SOAP *de novo* version 2.04 [parameters: -d 1 -M 3 -R -u -F; (23)] with K-mer values (24), and only the scaffolds with the longest N50 values were used. The clean reads were mapped against the scaffolds using SOAPaligner (parameters:-u−2 -m 200). The unaligned reads from each sample were collected to co-assemble with the same assembly parameters. The scaftigs larger than 500-bp were used for gene prediction by MetaGeneMark with -p 0 option (25). We also generated a non-redundant gene catalog by using cd-hit-est v4.6.6 [parameters: -G 0 -aS 0.9 -g 1 -d 0 -c 0.95; (26)], which included a greedy incremental clustering algorithm and the criteria of identity >95% and overlap >90% of the shorter genes.

Taxonomic assignments of protein sequences were analyzed using DIAMOND v0.8.28.90 (parameters: diamond blastp –evalue 10 –max-target-seqs 250) alignment against the NCBI-NR database by CARMA3 [parameters: carma –classify-blast –type p –database p; (27)]. The functional assignments of protein sequences were obtained on the basis of DIAMOND (default parameters except that -k 50 -sensitive -e 0.00001) alignment against the KEGG protein database release 79 (28). The relative abundance of genes for each sample was quantified by calculating the number of aligned reads using SOAP2 (parameters: -m 200 -x 400 -s 119). The relative abundance of microbial taxa was calculated by summing the abundance of the respective genes belonging to each category per sample and were based on the taxonomic assignments. We also summarized the relative gene abundance profile in KEGG functional profiles. The alpha diversity (Shannon index and Richness) was analyzed to estimate microbial diversity. The overall differences in microbial community structures in the HED and ND groups were visualized by principal component analysis (PCA) based on genus distribution.

The quality control of raw reads was performed to remove artificial replicates, host sequences, and low-quality reads. To obtain high quality clean reads, the residual rRNA sequences were excluded by alignment of rRNA, tRNA, and the SILVA database (29), and the remaining mRNA sequences were used for *de novo* assembly by Trinity v0.27 (30). A non-redundant gene catalog was obtained by integrating all sequences and removing redundant sequences using cd-hit-est (the criteria of the sequence consistency >95% and overlap >90% of the shorter genes). For taxonomic assignments, the assembled mRNA sequences were aligned to the NCBI-NR database using DIAMOND (parameters: diamond blastp –evalue 10 –max-target-seqs 250). Functional annotations were generated using DIAMOND against KEGG databases. The relative abundances of gut microbiota and their potential functional profiles were calculated. The PCA was used to visualize microbial community structures in the HED and ND groups based on genus distribution.

### Host RNA Extraction and Transcriptome Sequence Analysis

The liver and abdominal aorta tissues were collected from the HED and ND groups. Samples were immediately snap-frozen in liquid nitrogen and stored at −80°C. Particularly, we selected the representative specimens of atherosclerotic tissue in abdominal aorta for transcriptome analysis. Total RNA was extracted from these tissues using the TRIzol reagent (Invitrogen, Shanghai, China), and treated with RNase-free DNase I (TaKaRa, Shanghai, China) to remove residual genomic DNA. RNA purity and integrity were ensured and RNA libraries and sequencing analysis were performed accordance with the metatranscriptome sequence analysis. Reads that passed quality control were aligned to the *Sus scrofa* genome (https://www.ncbi.nlm.nih.gov/genome/?term=pig) using Tophat 2.0.10 with the default parameters (31), and only reads uniquely aligned to known genes were used for further analysis. The Cufflinks version 2.1.1 (31) was used to assemble annotated mRNAs transcripts individually with the default parameters. The conservation levels for mRNAs were evaluated using their 8-way PhastCons score (32). The expression levels of mRNAs were calculated by FPKM (Fragments per kilobase of transcript per million mapped reads) using Cuffquant version 2.1.1 (31). The differentially expressed mRNAs between the HED and ND groups were identified by the Bayes-regularized *t*-test with an FDR correction using Cyber-T bayesreg. The FDR <0.05 and |log_2_ (fold change)| >1 were considered statistically significant. In order to validate the RNA-seq results, qPCR was performed as described above. A total of eight DEGs (*CMPK2, CXCL10, MX2, DDX60, RSAD2, SPP1, DHX58*, and *PLAC8*) were selected. The primers for the specific genes and the housekeeping gene 18S rRNA used for normalization can be found in Supplementary Table 2. To identify significantly enriched GO terms and the main biological pathways associated with DEGs, we conducted GO and KEGG enrichment analyses using DAVID (database for annotation, visualization, and integrated discovery) v.6.8 (33). The significant GO terms and KEGG pathways were set to *P* < 0.05 and molecule number >2.

### Metabolome Profiling

The ileal contents (*n* = 17) and liver (*n* = 20) and abdominal aorta (*n* = 16) tissues were collected form minipigs, and analyzed by untargeted liquid chromatography-mass spectrometry (LC-MS). Samples were diluted with 300 mL chromatographic acetonitrile and mixed by ultrasonic disruption for 10 min and the vortexed. The mixture was then centrifuged at 12,000 rpm and 4°C for 15 min to remove any sediment. The metabolites were separated by the Waters Acquity UPLC I CLASS system (Waters, Milford, MA) with an HSS T3 column (4.6 × 100 mm, 3 um, Waters U.K.) at 40°C. Solvent A (water with 0.1% formic acid) and solvent B (acetonitrile with 0.1% formic acid) were used as the gradient elution. The gradient used was: 0–12 min linear gradient from 5 to 95% B; 12–17 min from 95 to 5% B in order for the column to re-equilibrate before the next injection. The experimental procedure and data acquisition for analysis were carried out according to a previous study (34).

The raw data were collected using SIEVE software (Thermo) processed for peak area calculation and peak normalization, and then filtered to identify potential discriminant variables. The exported data were processed using multivariate data analysis by SIMCA-P 14.0 software (Umetrics AB, Umea, Sweden). The OPLS-DA model was used in data processing of metabolomics, and the obtained score plot was used to establish a model visualization. Under circumstances of combining VIP > 1 (variable importance in the projection) and *P* < 0.05 (*t*-test for filtered data), molecular structures could be further identified.

### Statistical Analysis

All data are presented as means ± SEM, and the student's *t*-test was used to compare between groups. Only microbial taxa with a relative abundance higher than 0.01% in at least 50% of individuals were used for further analysis. Principal component analysis (PCA) was conducted based on the normalization using –log_10_(value) of the relative abundance of microbial taxa. Metastats analysis was used to determine the significantly differentially expressed gut microbiota and potential functional profiles between the HED and ND groups, and *P* < 0.05 or FDR <0.2 were considered to be statistically significant.

## Results

### Atherosclerotic Development in the MetS Model

**Figure 1:**
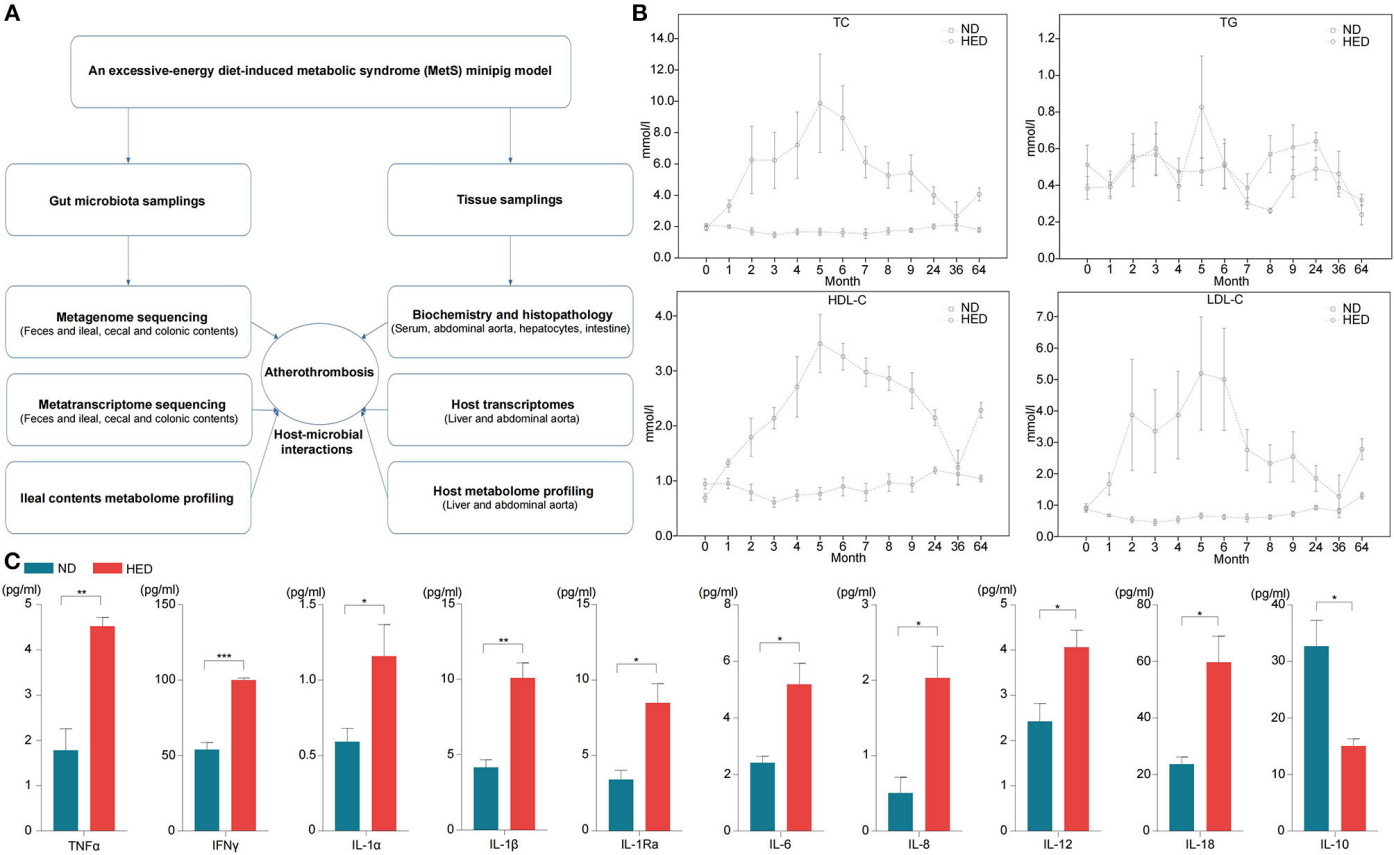
**(A)** Flow chart depicting gut microbiota and the tissue sampling process in the MetS model. **(B)** Levels of the indicated serum lipids, monitored over the course of the 64-month experiment (0–9th months: *n* = 22; 24–64th months: *n* = 12). **(C)** Levels of the indicated plasma inflammatory factors in animals of the HED and ND groups (64-month experiment; *n* = 3–4 in each group). HED, high-energy diet; ND, normal diet. Data are shown as the mean ± SEM. *P < 0.05, **P < 0.01, ***P < 0.001, based on student's *t*-test.

To study the influence of diet-induced gut microbiota dysbiosis on atherothrombosis, we established a Wuzhishan minipig model that included 64 months of excessive energy dietary intervention (Figure 1A). As expected, the high-energy diet (HED) group had significantly increased body weight compared with the normal diet (ND) (standard chow diet) group (Supplementary Figure 1A). Compared with the ND group, **s**erum TC, HDL-C, and LDL-C levels were dramatically altered in the HED group (Figure 1B). Of note, serum LDL-C levels showed the largest increase in the HED group, reaching peak levels that were five times higher than that of individuals in the ND group after the 5th month of dietary intervention (Figure 1B). Serum TG in the HED group after 5 month of dietary intervention was also approximately two times higher than that of individuals in the ND group (Figure 1B). However, the ND group had relatively stable lipid and cholesterol levels during the 64 months dietary experiments (Figure 1B). Meanwhile, serum levels of pro-inflammatory cytokines including TNFα, IFNγ, IL-1α, IL-1β, IL-1Ra, IL-6, IL-8, IL-12, and IL-18 were significantly higher (*P* < 0.05) in the HED group than those in the ND group (Figure 1C). However, the serum level of the anti-inflammatory cytokine IL-10 was lower in the HED group compared to that in the ND group (Figure 1C). These findings suggested that MetS in the porcine model was induced by diets with excessive energy levels in the HED groups.

**Figure 2:**
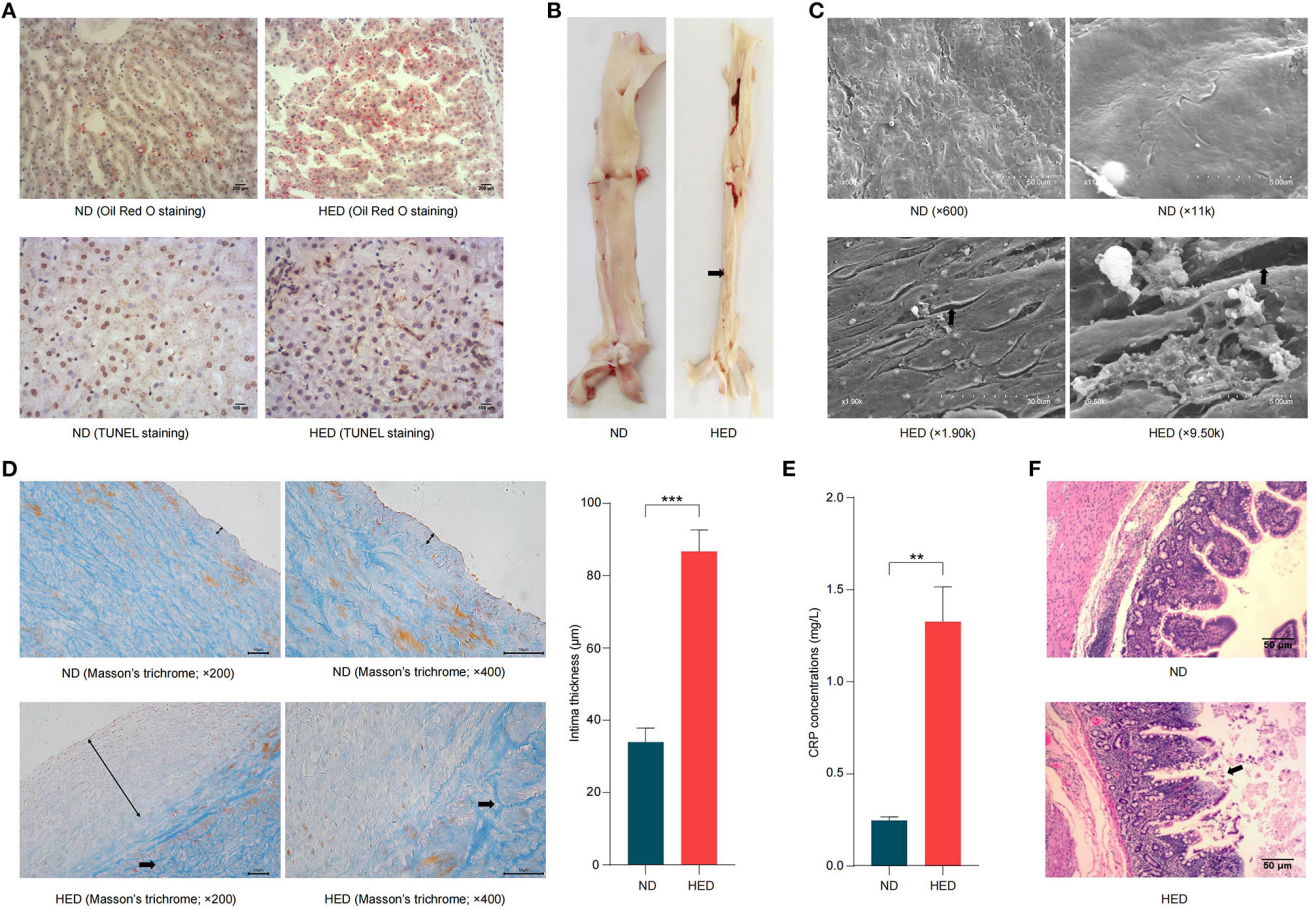
High-caloric load induced by excessive energy intake resulted in atherothrombosis in a metabolic syndrome (MetS) model. **(A)** Characterization of hepatocytes with Oil Red O staining (bars, 200 μm) and TUNEL (bars, 100 μm). **(B)** Representative images of white atheromatous plaque of abdominal aorta in the HED and ND group animals. The arrows pointed at the atheromatous plaque. **(C)** Characterization of abdominal aorta cross-sections (scanning electron micrographs). The arrows pointed at the endothelium denudation. **(D)** Characterization of abdominal aorta cross-sections based on Masson's trichrome staining (bars, 50 μm). Double-headed black arrows show intimal thickness, and single-headed black arrows pointed at the thicker collagen fibers. **(E)** Comparison of the CRP concentrations in the HED and ND groups. **(F)** H&E staining of ileal segments sections to detect lesions (bars, 50 μm). The arrows pointed at the necrosis and shedding of intestinal mucosal intraepithelial cells and naked lamina propria. Data are shown as the mean ± SEM. **P < 0.01, ***P < 0.001, based on student's *t*-test.

We next systematically investigated the histopathological changes in liver and abdominal aorta. The HED group showed significantly increased liver weight compared with the ND group (Supplementary Figure 1A). In hepatocytes, accumulated lipid droplets were observed by Oil Red O staining, indicating moderate hepatocytes steatosis in the HED group (Figure 2A). Moreover, TUNEL staining showed that apoptosis in hepatocytes was markedly increased in the HED group compared with the ND group (Figure 2A). In the HED group animals, abdominal aorta not only showed the decreased elasticity of blood vessel but also were observed white atheromatous plaque with a small amount of thrombus from top to bottom of vascular intima (Figure 2B). Scanning electron microscopy examination revealed that the HED group animals exhibited endothelium denudation, endothelial disorganization, and thrombogenesis in the abdominal aorta, which had a closed relationship with atherosclerotic development (Figure 2C). Histologic examination of the abdominal aorta showed the significantly thickened (*P* < 0.001) intima, and thicker collagen fibers (blue) in tunica media with disordered arrangement in the HED group compared to the ND group (Masson's trichrome staining; Figure 2D). The accumulation of extracellular lipids developed with the transition from intima to tunica media in the HED group (Oil Red O staining; Supplementary Figure 1B). Further, the CRP concentrations were significantly higher (*P* < 0.05) in HED group than those in ND group, which was potentially associated with the occurrence of atherosclerosis (Figure 2E). Taken together, our results suggested that the MetS porcine model, induced by diets containing excessive energy, promoted the onset of atherothrombosis.

### Impaired Intestinal Epithelial Integrity in the MetS Model

The formation and maintenance of tight junctions between intestinal epithelial cells is essential for maintaining barrier function and regulation intestinal permeability (35). In order to investigate the effects of a long-term, high-energy diet on the intestinal epithelial integrity, the mRNA expression levels of tight junction genes, *Occludin*, zonula occludens-1 (*ZO-1*), *Claudin*, and *E-Cadherin*, were determined along the length of gastrointestinal tracts (i.e., duodenum, ileum, cecum, colon, and rectum). The expression levels of tight junction genes (duodenum: *Occludin, ZO-1*, and *E-Cadherin*; ileum: *Occludin, ZO-1, Claudin*, and *E-Cadherin*; cecum: *Occludin* and *Claudin*; colon: *Claudin*; rectum: *Occludin* and *ZO-1*) were significantly lower (*P* < 0.05) in the HED group than those in the ND group (Supplementary Figure 1C). Of note, in ileal segment from the HED group, the expression levels of these tight junction genes were markedly lower than in other indicated intestinal segments. Moreover, western blotting analysis also showed an decrease in Occludin protein levels in the HED group (Supplementary Figure 1D). Meanwhile, the reduced expression of Occludin protein in the ileal segment was observed by immunofluorescence in the HED group (Supplementary Figure 1E). To further assess the intestinal epithelial integrity, histology analysis of ileum segment was analyzed by H&E staining (Figure 2F). The appearance of mucosal injury and marked neutrophilic infiltration in the ileum were found in HFD-fed pigs. These results demonstrated that the down-regulated expression levels of intestinal tight junctions led to impairment of intestinal epithelial integrity, which is suggestive of increased intestinal permeability.

### Dysbiosis of Gut Microbiota in the MetS Model

Gut microbiota have been demonstrated to regulate their hosts metabolism by interacting with the host's signaling pathways (36, 37). Therefore, we examined the microbial metagenome and metatranscriptome of gastrointestinal tracts by sequencing (Supplementary Tables 3, 4). In the metagenomic and metatranscriptomic datasets, alpha diversity (Shannon index and Richness) was markedly lower in the HED group than in the ND group (Supplementary Figure 2), which indicated loss of gut microbial diversity. Comparison of the HED group with the ND group further revealed that differences were detected in the indicated intestinal microbial community structures as shown by the results of PCA at the genus level (Supplementary Figure 3).

**Figure 3:**
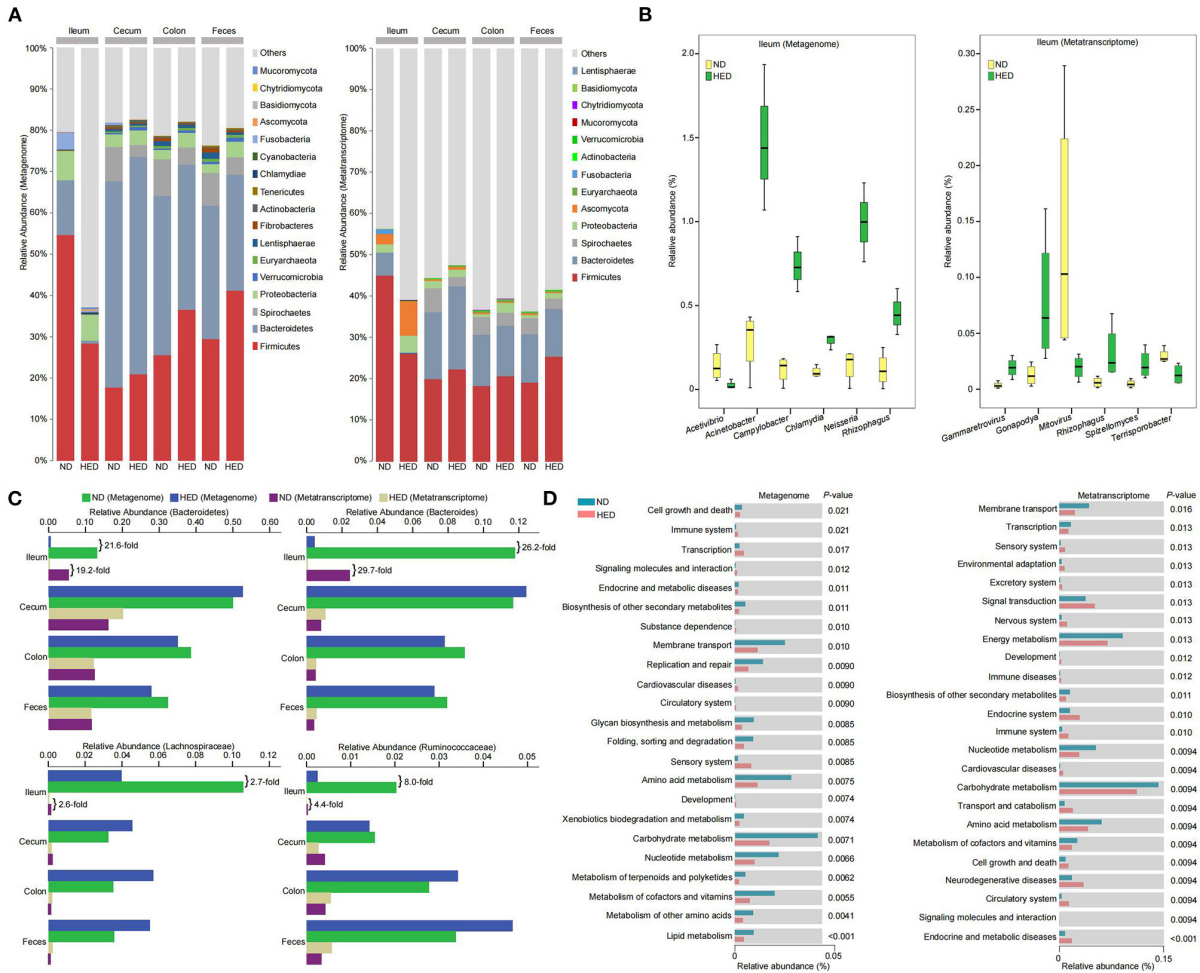
Microbial community composition and transcriptional activity along gastrointestinal tracts were altered in a MetS model. **(A)** The relative abundances of gut microbiota at phylum level in the indicated intestinal segment contents and fecal metagenome and metatranscriptome datasets between animals of the HED and ND groups (64-month experiment). **(B)** Significant differences (*P* < 0.05; metastats analysis) in the relative abundances of microbial genera in ileal contents detected between the HED and ND groups based on the metagenome and metatranscriptome datasets. **(C)** The relative abundances of butyrate-producing microbiota detected between the HED and ND groups based on the metagenome and metatranscriptome datasets. **(D)** Differences in the relative abundances of KEGG functional categories (level 2) in the ileal microbiota between the HED and ND groups based on the metagenome and metatranscriptome datasets.

The species with relative abundance higher than 0.01% in at least 50% of individuals at the phylum level were selected to generate a columnar summation diagram of species relative abundance (Figure 3A). Five major phyla at the level of DNA and RNA in the ileal segment consisted mainly of Firmicutes, Proteobacteria, Bacteroidetes, Mucoromycota, and Ascomycota, and the relative abundances of Mucoromycota (DNA level: 0.57 vs. 0.14%, RNA level: 0.15 vs. 0.03%) and Ascomycota (DNA level: 0.19 vs. 0.06%, RNA level: 8.21 vs. 2.47%) were much higher in the HED group than those in the ND group (Figure 3A). The dominating bacterial phyla at the level of DNA and RNA in the large intestine consisted mainly of Firmicutes, Bacteroidetes, and Proteobacteria. The Firmicutes to Bacteroidetes ratio (feces: 1.48 vs. 0.91 [DNA level], 2.19 vs. 1.63 [RNA level]) and the relative abundance of Proteobacteria (cecum: 3.52 vs. 3.09% [DNA level], 1.85 vs. 1.73% [RNA level]; colon: 3.54 vs. 2.22% [DNA level], 2.34 vs. 0.77% [RNA level]; feces: 3.81 vs. 2.17% [DNA level], 1.29 vs. 0.75% [RNA level]) were much higher in the HED group than in the ND group (Figure 3A).

A metastats analysis identified dominant microbiota that accounted for the greatest differences observed between the HED and ND groups (Supplementary Table 5). In the ileal segment, five genus-level taxa (i.e., *Neisseria, Acinetobacter, Campylobacter, Chlamydia*, and *Rhizophagus*) at the DNA level were 2- to 10-fold enriched in the HED group, while the abundance of *Acetivibrio* was significantly higher (*P* < 0.05) in the ND group (Figure 3B and Supplementary Table 5). At the RNA level, four genus-level taxa (i.e., *Rhizophagus, Gonapodya, Spizellomyces*, and *Gammaretrovirus*) were significantly enriched (*P* < 0.05) in the HED group, while two genus-level taxa (i.e., *Terrisporobacter* and *Mitovirus*) were significantly enriched (*P* < 0.05) in the ND group (Figure 3B and Supplementary Table 5). Of particular note, the abundances of SCFAs-producing bacteria at the DNA and RNA levels in the ileal segment, including phylum Bacteroidetes, genus *Bacteroides*, and family Lachnospiraceae and Ruminococcaceae, were 2- to 30-fold reduced in the HED group compared with the ND group (Figure 3C). We found genus-level taxa at the DNA level, including *Saccharicrinis, Parabacteroides*, and *Sutterella* in cecal segment and *Roseburia, Eubacterium*, and *Butyrivibrio* in colonic segment, were all significantly enriched (*P* < 0.05) in the HED group, while in the ND group, *Treponema* and *Sphaerochaeta* in cecal segment and *Alistipes* and *Treponema* in colonic segment, were significantly enriched (*P* < 0.05; Supplementary Figure 4 and Supplementary Table 5). The abundances of *Lactobacillus* and *Batrachochytrium* in cecal segment and *Lipomyces* in colonic segment at the RNA level were higher in the HED group than in the ND group (Supplementary Figure 4 and Supplementary Table 5), while five genera (i.e., *Treponema, Turicibacter, Sphaerochaeta, Cellulosilyticum*, and *Acetobacter*) in cecal and three genus-level taxa (i.e., *Mitovirus, Alistipes, and Bacillus*) in colonic segments were relatively depleted in the HED group (Supplementary Figure 4 and Supplementary Table 5). In feces, we found three genera at the DNA level, including *Roseburia, Eubacterium*, and *Butyrivibrio*, were significantly enriched (*P* < 0.05) in the HED group, whereas in the ND group, three genera, including *Alistipes, Treponema*, and *Fibrobacter*, were significantly enriched (*P* < 0.05; Supplementary Figure 4 and Supplementary Table 5). At the RNA level, the abundance of *Campylobacter* was higher in the HED group than in the ND group, while four genera including *Mitovirus, Fibrobacter, Lachnoclostridium*, and *Coprobacillus* were relatively depleted in the HED group (Supplementary Figure 4 and Supplementary Table 5).

The dominant KEGG functional categories (level 2) of microbiota at the level of DNA and RNA in intestinal segments included metabolism, genetic information processing, environmental information processing, and cellular processes (Figure 3D and Supplementary Figure 5). In the ileal microbiome, the functional categories (level 3) were significantly differentially abundant (*P* < 0.05) between the HED and ND groups (Supplementary Table 6). Of these categories, arachidonic acid, cytochrome P450, sphingolipids, porphyrin, chlorophyll, arginine, proline, pantothenate, CoA, aminoacyl-tRNA, valine, leucine, and isoleucine metabolism in the HED group were significantly up-regulated (*P* < 0.05) compared to the ND group. In the large-intestinal microbiome, we identified functional categories (level 3) with significant differential abundance (*P* < 0.05) between the HED and ND groups (Supplementary Tables 7–9). Of these categories, the metabolism of terpenoids, polyketides, taurine, hypotaurine, propanoate, pyruvate, thiamine, butanoate, and aminobenzoate in the HED group were significantly up-regulated (*P* < 0.05) compared to the ND group. Taken together, these data demonstrated that harmful bacteria were enriched while probiotics were dramatically down-regulated, which in turn had a profound influence on intestinal homeostasis and further affected host metabolism in the MetS model.

### Global Overview of Liver and Abdominal Aorta Transcriptomes in the MetS Model

**Figure 4:**
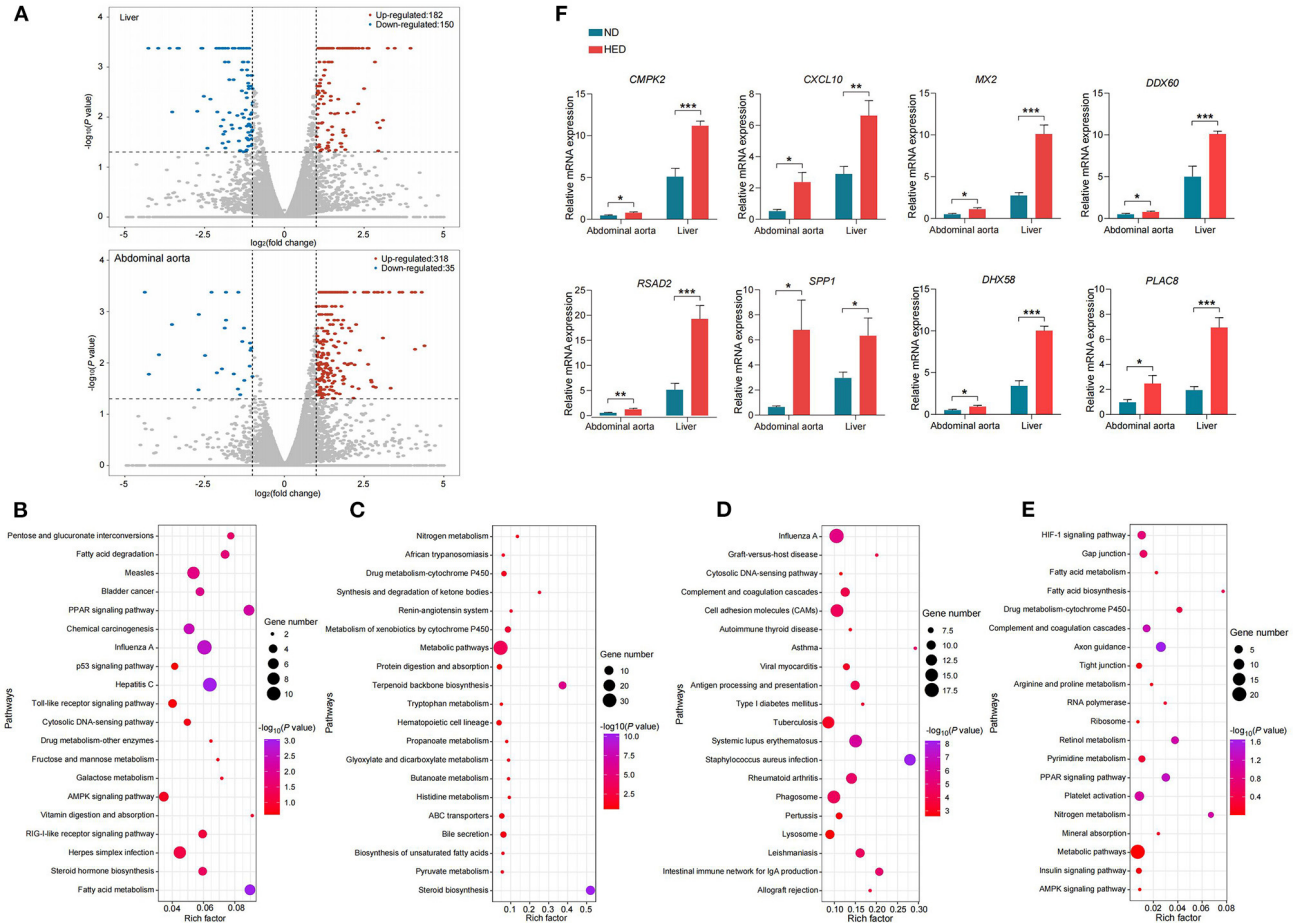
Altered gene expression of liver and abdominal aorta in a MetS model. **(A)** Volcano plots showing genes with decreased (blue dots) and increased (red dots) expression of mRNAs from liver and abdominal aorta in the HED group compared with the ND group. **(B)** The top 20 KEGG pathways (elevated levels) in liver. **(C)** The top 20 KEGG pathways (decreased levels) in liver. **(D)** The top 20 KEGG pathways (elevated levels) in abdominal aorta. **(E)** The top 20 KEGG pathways (decreased levels) in abdominal aorta. **(F)** qPCR confirming the RNA-seq data for the indicated common liver/abdominal aorta DEGs detected in the comparison of the HED and ND groups (64-month experiment; *n* = 4 in each group). Data are shown as the mean ± SEM. *P < 0.05, **P < 0.01, ***P < 0.001, based on student's *t-*test.

Liver can affect cholesterol balance through modulating the secretion of bile acids (38). In order to thoroughly investigate the potential interaction between liver and abdominal aorta in developing atherogenesis in the MetS model, we performed next-generation RNA sequencing from the two tissues (Supplementary Table 10), in both the HED and ND groups. There were 332 (182 up-regulated and 150 down-regulated) and 353 (318 up-regulated and 35 down-regulated) differentially expressed genes (DEGs) identified between the HED and ND groups in liver and abdominal aorta [FDR <0.05 and |log_2_(fold change)| > 1, Figure 4A], respectively.

GO enrichment and KEGG pathway analysis of the DEGs in liver detected significantly enriched (*P* < 0.05) GO categories and pathways (Figures 4B,C and Supplementary Table 11), respectively. The significant GO categories and pathways highlighted main functional categories: (1) immune and inflammatory response; (2) lipid and steroid metabolism; (3) inorganic ion transport and metabolism. In abdominal aorta, we also identified significantly enriched (*P* < 0.05) GO categories and KEGG pathways, respectively (Figures 4D,E and Supplementary Table 12). Of these GO categories and pathways, the biological processes identified were significantly associated with inflammatory response and lipid metabolism. To confirm the sequencing results, we performed qPCR for eight DEGs (*CMPK2, CXCL10, MX2, DDX60, RSAD2, SPP1, DHX58*, and *PLAC8*) in both liver and abdominal aorta. Consistent with sequencing results, all genes were found to be differentially expressed between the HED and ND groups (*P* < 0.05; Figure 4F).

### Metabolome Profiling Revealed Perturbative Arachidonic Acid Metabolism Associated With Atherothrombosis in the MetS Model

**Figure 5:**
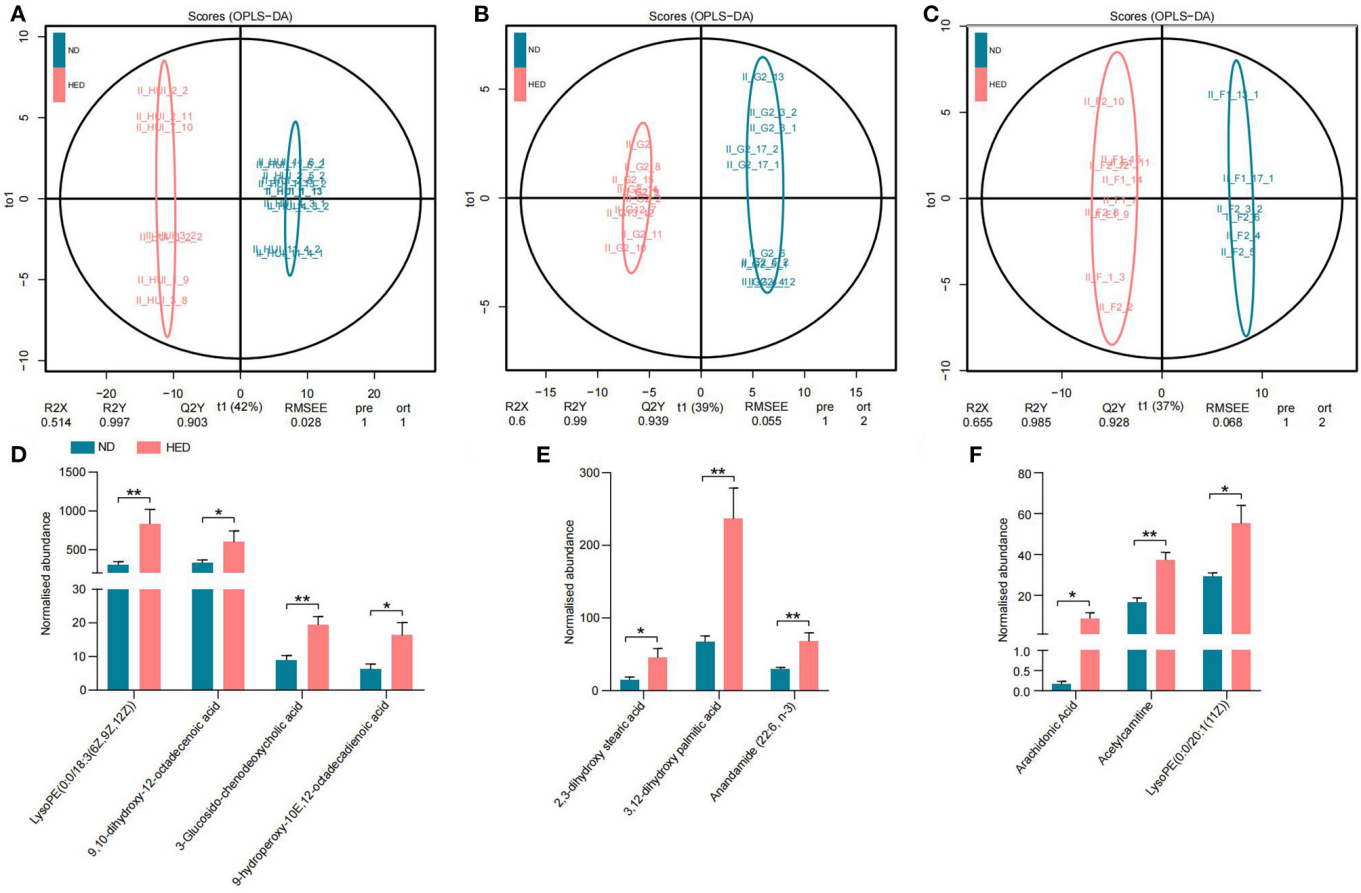
Modulated arachidonic acid metabolism led to atherothrombosis in a MetS model. **(A)** OPLS-DA plot in the ileal contents. **(B)** OPLS-DA plot in the liver. **(C)** OPLS-DA plot in the abdominal aorta. **(D–F)** Comparison of the normalized abundance of metabolites associated with the pathway of arachidonic acid metabolism in the HED and ND groups in the ileal contents, liver, and abdominal aorta tissues, respectively. Data are shown as the mean ± SEM. *P < 0.05, **P < 0.01, based on student's *t*-test.

In order to further investigate whether the gut microbiota dysbiosis was closely connected with atherothrombosis, we examined the untargeted metabolome profiles generated from ileal contents and liver and abdominal aorta samples using liquid chromatography-mass spectrometry (LC-MS). We obtained positive (ESI+) and negative (ESI-) mode TIC chromatograms obtained from the HED and ND group animals (Supplementary Figure 6). The orthogonal projection to latent structure discriminant analysis (OPLS-DA) model was used to identify differential metabolites between the HED and ND groups. The OPLS-DA model provided excellent fits and was highly predictive in the ESI+ the ESI– modes (Figures 5A–C). We found target metabolites with VIP > 1 and *P* < 0.05 (*t*-test) following screening and the discovery of biomarkers, and the chemical structures of the metabolites were identified according to online databases (e.g., Metlin; http://metlin.scripps.edu).

**Figure 6:**
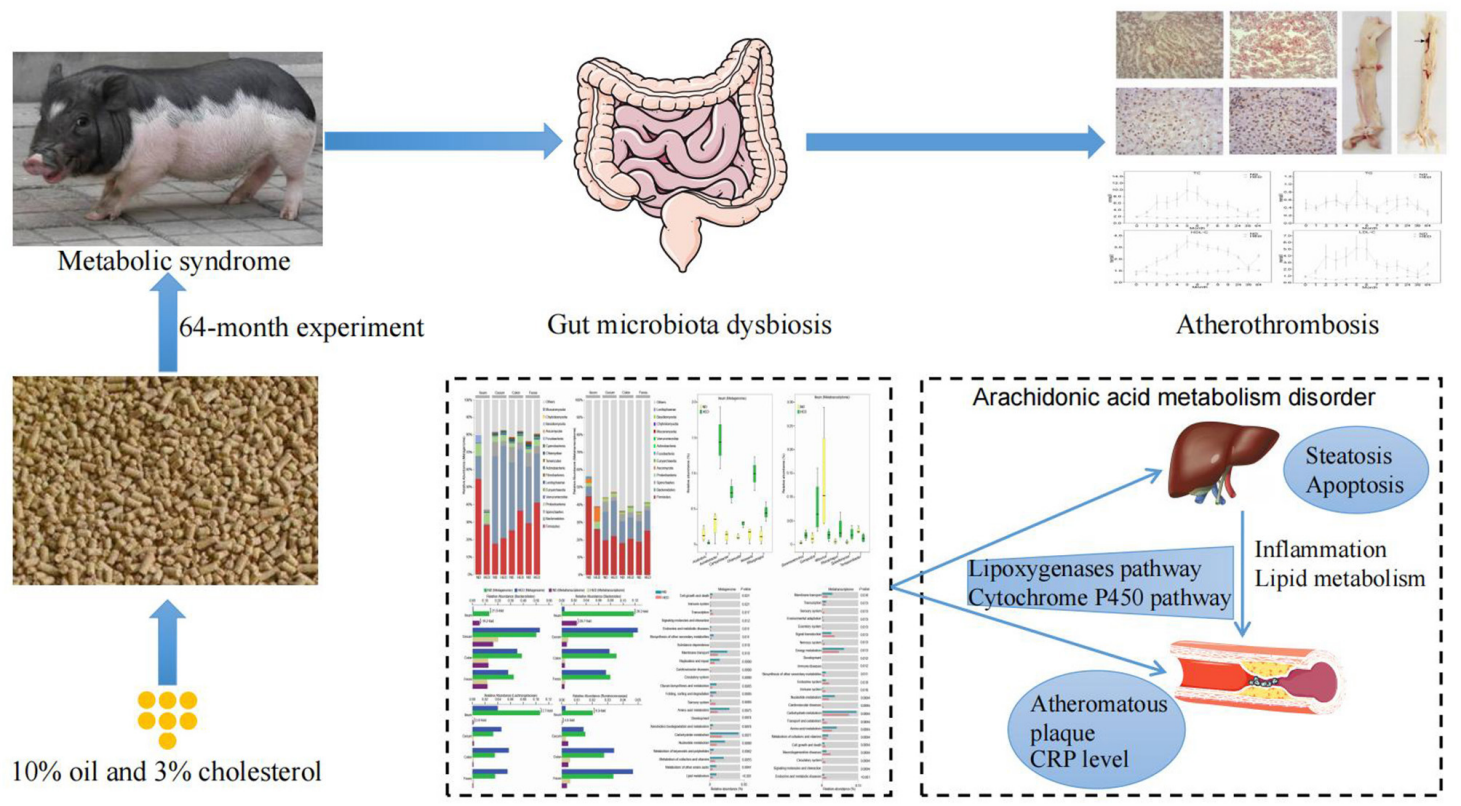
Schematic diagram of the proposed mechanism of arachidonic acid metabolism-induced atherothrombosis via gut microbiota dysbiosis in a MetS model.

Diets with excessive energy levels were sufficient to trigger widespread changes in metabolites. For example, 215 metabolites in the ileal contents were obtained from comparative analysis of the two groups, including 93 metabolites in the ESI+ mode and 122 metabolites in the ESI– mode (Supplementary Figure 7A). Meanwhile, 105 metabolites (53 in the ESI+ mode and 52 in the ESI– mode) in the liver (Supplementary Figure 7B) and 111 metabolites (31 in the ESI+ mode and 80 in the ESI– mode) in the abdominal aorta (Supplementary Figure 7C) were significantly different between the HED and ND groups. Biomarkers from the ileal contents were elevated in the HED group and included LysoPE [0:0/18:3 (6Z, 9Z, 12Z)], 9,10-dihydroxy-12-octadecenoic acid, 3-Glucosido-chenodeoxycholic acid and 9-hydroperoxy-10E,12-octadecadienoic acid (Figure 5D), which belong to the pathway of arachidonic acid metabolism and could cause inflammation and lipid infiltration. Additionally, the HED group displayed elevated levels of 2,3-dihydroxy stearic acid, 3,12-dihydroxy palmitic acid and Anandamide (22:6, n-3) in the liver (Figure 5E) and LysoPE [0:0/20:1 (11Z)], Acetylcarnitine and Arachidonic Acid in the abdominal aorta (Figure 5F). These significant metabolites highlight the critical functional category, i.e., arachidonic acid metabolism regulation, impacted by high caloric diets. We also examined metabolites of gut microbes, i.e., LPS. However, we found that LPS showed no significant difference between the two groups (Supplementary Figure 7D). These results indicated a correlation between the pro-inflammatory role of arachidonic acid metabolism associated with atherothrombosis and gut microbiota dysbiosis in the MetS model (Figure 6).

## Discussion

MetS is a major risk factor for atherosclerotic cardiovascular disease, which is the most common cause of death worldwide and is most prevalent in developed countries. Atherothrombosis in MetS is commonly accompanied by gut microbiota dysbiosis, and therefore its underlying molecular mechanisms is worth investigation. We conducted a detailed phenotypic assessment of atherothrombosis from a MetS model induced by an excessive energy diet and revealed a notable elevation in the level of pro-inflammatory cytokines and a visible atherosclerotic lesion in abdominal aorta. Furthermore, disruption of intestinal epithelial integrity was observed in the MetS model. Notably, the long-term overconsumption of excessive energy altered the local gut microbiota composition and transcriptional activity with significant increases in pro-inflammatory intestinal bacteria alongside significant decreases in anti-inflammatory intestinal bacteria. Also, we identified the pathways presented in liver and abdominal aorta associated with lipid and steroid metabolism and inflammatory response. Lastly, our study uncovered the pivotal role of the arachidonic acid metabolism pathway in the host-gut microbial metabolism axis.

Atherosclerosis is regarded as a major risk factor for cardiovascular diseases that are the most common cause of death worldwide especially in developed countries (39). The development of atherosclerosis is a continuous process, which typically starts with deposition of lipids in intima and subsequently endothelial activation and infiltration of inflammatory cells into sub endothelial layer (2). Particularly, advanced atherosclerosis, characterized by accumulation of apoptotic foam cells leading to the formation of necrotic lipid cores, is the major cause of severe stenosis and acute thrombotic events (3).

Accumulating evidences suggested that gut microbiota dysbiosis could lead to an increased risk of developing atherogenesis through the modulation of pro-inflammatory responses (40, 41). In our study, the metagenome data combined with metatranscriptome data showed that the local intestinal microbiota had significant differences between the HED and ND groups. In the ileal segment, the relative abundance of the fungal community included *Rhizophagus, Spizellomyces*, and *Gonapodya* were significantly enriched in the HED group. Recent studies have revealed that these fungal genera, previously found to be involved in the regulation of microbial immune homeostasis, may be associated with pro-inflammatory responses (42–44). Significantly, a drastic decrease in a group of SCFAs-producing bacteria including phylum Bacteroidetes, genus *Bacteroides*, family Lachnospiraceae, and Ruminococcaceae was observed in the HED group. Decreased butyrate correlates with gut barrier dysfunction (45), which increases the permeability of the gut barrier to endotoxin molecules such as lipopolysaccharides (LPS). In the ileal segment, the significant KEGG categories were mainly involved in metabolism of arachidonic acid, sphingolipid, porphyrin and chlorophyll, cytochrome P450, pantothenate and CoA, and amino acids. Furthermore, a high energy diet can induce an imbalance in the large intestinal microbiota, especially an increase in the abundance of endotoxin-producing Enterobacteriaceae which plays a crucial role in the synthesis of the O-antigen of LPS (46). However, SCFA-producing bacteria *Treponema* and *Sphaerochaeta* were also reduced in the HED group. Of note, the significantly abundant functional categories were mainly involved in bacterial chemotaxis, bacterial secretion systems, and biotin and linoleic acid metabolism in large intestinal microbiota. In summary, this study found that a diet-induced structural imbalance in intestinal microbiota was closely related to inflammation and the subsequent development of atherosclerosis.

Transcriptome analysis demonstrated that the HED group animals underwent a change in the expression of 332 genes in liver and 353 genes in abdominal aorta, which are mainly involved in lipid metabolism and inflammatory response. Studies reported that hepatic cholesterol synthesis-related proteins, such as sterol regulatory element-binding protein 1, fatty acid synthase, and acetyl-CoA carboxylase, elevated levels of serum lipids (47). Our RNA-sequencing results showed that gene expression of *CYP1A2, CYP3A46*, and *CYP3A88* was upregulated. These genes are critical rate-limiting enzymes for bile acid synthesis from cholesterol, which is the primary route of cholesterol excretion (48). Accumulating evidence has indicated that changes in serum lipid metabolism are well-recognized in the pathogenesis of atherosclerosis (49). Pathway analysis revealed that fatty acid metabolism and PPAR signaling pathways were among the top pathways in liver. Upregulation of PPAR signaling pathway has been observed in lipid and carbohydrate metabolism and inflammation status. Our data showed that HED diet induced the expression of certain mRNA pattern recognition receptors, such as Toll-like receptors and RIG-I-like receptors, which participate in the recognition of bacteria and viruses in liver (50). Imbalance of the intestinal microbiota can affect dysfunction of the intestinal mucosal barrier, which lead to increased permeability of gut barrier to endotoxins, pathogenic bacteria and other antigens. So, Toll-like and RIG-I-like receptor signaling pathways are activated by bacteria and viruses to induce pro-inflammatory mediators. Moreover, The various functions of these genes [*ABCG1, CXCL10, LGALS3, OAS1, OAS2, TOP2A, ISG12(A), ISG15*, and *TRPV2*] in abdominal aorta implied strong impacts of cyclooxygenases and cytochrome P450 activity on the arachindonic acid metabolism signaling pathway. Our KEGG functional analyses of DEGs in abdominal aorta highlighted the enrichment in the HED group of proinflammatory-associated pathways such as *Staphylococcus aureus* infection, intestinal immune network for IgA production and cell adhesion molecules. These results suggested that the transcriptome alterations in liver and abdominal aorta exacerbated the progression of atherosclerosis via regulating lipid metabolism and inflammatory pathways.

Gut microbiota have been considered a metabolic organ, which enhances the host's metabolic capacity for processing nutrients and regulates the activities of multiple pathways in a variety of organ systems. We further performed host-gut microbial metabolic interactions to further explore the source of the inflammation. Metabolomics profiling data suggested that the elevated levels of metabolites associated with the pathway of arachidonic acid metabolism were detected in both host (liver and abdominal aorta) and gut microbiota in the MetS model. Arachidonic acid, a major long-chain n-6 polyunsaturated fatty acid and its derivatives are involved in inflammation and coagulation (51). It is well-established that arachidonic acid could be oxidatively modified by three pathways: lipoxygenase to leukotrienes; cyclooxygenases to prostaglandins and thromboxanes; cytochrome P450s to hydroxyeicosatetraenoic and hydroxyoctadecadienoic acids (52). Recently, studies have outlined the relationship between arachidonic acid metabolism and atherothrombosis (34, 53). These results indicated that metabolic disorders caused arachidonic acid metabolic dysfunction and exacerbated the progression of atherosclerosis.

The minipig is more similar to humans in arterial physiology and anatomy, especially with regard to the formation process of atherosclerotic lesions compared with rodents such as mouse (24, 54). In our study, the inbred Wuzhishan minipigs are particularly suitable for cardiovascular disease research, due to their reasonable size, early maturity as well as their inter-individual similarity. Clinical studies showed that excessive energy intake in this model could lead to the development of human-like atherosclerosis (13). In our study, the gut microbiome in the MetS model had a higher taxonomic and functional overlap with the human gut microbiome, implying that the gut microbiome could be predictive of results in humans (see Supplementary Material).

## Conclusions

Our findings suggested that the modulation of gut microbiota altered arachidonic acid metabolism and further affected host liver and abdominal aorta transcriptome profiles. As a result, the abnormal metabolism of arachidonic acid played a critical role in pro-inflammatory response in the host-gut microbial metabolism axis, resulting in atherothrombosis in the MetS model. The results of this study advance our understanding of atherothrombosis induced by gut microbiota dysbiosis and may lead to future therapeutic strategies to combat this disease.

## Data Availability Statement

The datasets presented in this study can be found in online repositories. The names of the repository/repositories and accession number(s) can be found below: EBI Metagenomics (Accession No: PRJEB48889).

## Ethics Statement

The animal study was reviewed and approved by Animal Care and Use Committee of the Germplasm Resource Center of Chinese Experimental Minipigs. Written informed consent was obtained from the owners for the participation of their animals in this study.

## Author Contributions

KL and Y-LM conceived and designed the project. X-LZ, S-SL, G-MX, C-JX, Z-YF, KX, NW, YW, J-JC, and Z-GL collected the samples. S-SX, X-LZ, and S-SL analyzed the data and wrote the paper. All authors have reviewed and approved the final manuscript.

## Funding

This work was supported by the National Key Research and Development Program of China (2021YFF1000600), the National Natural Science Foundation of China (32072690), the Major Scientific Research Tasks for Scientific and Technological Innovation Projects of the Chinese Academy of Agricultural Sciences (CAAS-ZDRW202006), and the Agricultural Science and Technology Innovation Program of the Chinese Academy of Agricultural Sciences (ASTIP-IAS05).

## Conflict of Interest

The authors declare that the research was conducted in the absence of any commercial or financial relationships that could be construed as a potential conflict of interest.

## Publisher's Note

All claims expressed in this article are solely those of the authors and do not necessarily represent those of their affiliated organizations, or those of the publisher, the editors and the reviewers. Any product that may be evaluated in this article, or claim that may be made by its manufacturer, is not guaranteed or endorsed by the publisher.

## Supplementary Material

Supplementary Figure 1 **(A)** Comparison of the body weight and liver weight in the HED and ND groups. **(B)** Characterization of abdominal aorta cross-sections based on Oil Red O staining (bars, 200 μm). **(C)** The expression of tight junction genes including *Occludin, ZO-1, Claudin*, and *E-Cadherin* in tissues from five intestinal sections (duodenum, ileum, cecum, colon, and rectum) between the HED and ND groups (*n* = 4 in each group). **(D)** Intestinal protein expression of Occludin in the HED and ND groups. **(E)** Immunofluorescence analysis of the Occludin protein in the ileal segment (bars, 50 μm). The red signals are for Occludin; the blue signals are for DAPI (nuclear staining). The arrows pointed at the Occludin protein. Data are shown as the mean ± SEM. ^*^*P* < 0.05, ^**^*P* < 0.01, based on student's *t*-test.

Supplementary Figure 2 Comparison of microbial alpha diversity (Shannon index) and gene richness at the genus level between the HED and ND groups across the indicated intestinal contents and feces. ^*^*P* < 0.05, based on student's *t*-test (two-tailed).

Supplementary Figure 3 Principal component analysis based on genus abundances between the HED and ND groups in the metagenome and metatranscriptome datasets. The **(A)** metagenome and **(B)** metatranscriptome datasets of the ileal microbiome. The **(C)** metagenome and **(D)** metatranscriptome datasets of the cecal microbiome. The **(E)** metagenome and **(F)** metatranscriptome datasets of the colonic microbiome. The **(G)** metagenome and **(H)** metatranscriptome datasets of fecal sample microbiome. Red dot represents the HED group animals; black box represents the ND group animals.

Supplementary Figure 4 Significant differences (*P* < 0.05; metastats analysis) in the abundances of microbial genera detected between the HED and ND groups based on the metagenome and metatranscriptome datasets in the indicated intestinal contents and feces.

Supplementary Figure 5 KEGG functional categories (level 2) of **(A)** ileal, **(B)** cecal, **(C)** colonic, and **(D)** fecal microbiome in the metagenome and metatranscriptome datasets.

Supplementary Figure 6 Comparison of **(A)** ileal contents positive ion mode analysis, **(B)** ileal contents negative ion mode analysis, **(C)** liver positive ion mode analysis, **(D)** liver negative ion mode analysis, **(E)** abdominal aorta positive ion mode analysis, **(F)** abdominal aorta negative ion mode analysis in the HED and ND groups.

Supplementary Figure 7 Heatmap of untargeted ileal contents **(A)**, liver **(B)**, and abdominal aorta **(C)** tissues metabolomic analysis, showing the accumulation (abundance patterns) of the indicated annotated metabolites with significant changes (VIP > 1 and unpaired *t*-test *P* < 0.05) between the HED and ND groups (64-month experiment). **(D)** Quantification of serum LPS level (*n* = 3 in each group). Data were shown as mean ± SEM, based on student's *t*-test.

Supplementary Table 1 Primers for tight junction genes validations by qPCR.

Supplementary Table 2 Primers for the DEGs detected both in liver and abdominal aorta validations by qPCR.

Supplementary Table 3 Statistics of metagenomic sequencing across different intestinal segments.

Supplementary Table 4 Statistics of metatranscriptomic sequencing across different intestinal segments.

Supplementary Table 5 Significant differences (*P* < 0.05; metastats analysis) in the abundance of microbial genera in intestinal contents detected between the HED and ND groups based on the metagenome and metatranscriptome datasets.

Supplementary Table 6 The significantly differentially functional categories (level 3) at the DNA and RNA levels between the HED and ND groups in ileal microbiota.

Supplementary Table 7 The significantly differentially functional categories (level 3) at DNA and RNA levels between the HED and ND groups in the cecal microbiota.

Supplementary Table 8 The significantly differentially functional categories (level 3) at the DNA and RNA levels between the HED and ND groups in colonic microbiota.

Supplementary Table 9 The significantly differentially functional categories (level 3) at the DNA and RNA levels between the HED and ND groups in fecal microbiota.

Supplementary Table 10 Statistics of clean reads in liver and abdominal aorta.

Supplementary Table 11 Functional enrichment analysis of differentially expressed genes identified from liver mRNAs.

Supplementary Table 12 Functional enrichment analysis of differentially expressed genes identified from abdominal aorta mRNAs.
