# Supplementary material for: Multi-Omic Analysis in a Metabolic Syndrome Porcine Model Implicates Arachidonic Acid Metabolism Disorder as a Risk Factor for Atherosclerosis
Source: Front Nutr. 2022 Feb 23;9:807118. doi: 10.3389/fnut.2022.807118 (PMC8906569; doi:10.3389/fnut.2022.807118)
Supplement: Supplementary file 1 [file Presentation_1.pdf]

Multi-omic analysis in a metabolic syndrome porcine model implicates arachidonic acid metabolism disorder as a risk factor for atherosclerosis

Song-Song Xu[1,2,†], Xiu-Ling Zhang[1,3,†], Sha-Sha Liu[1,4,†], Shu-Tang Feng[1], Guang-Ming Xiang[1], Chang-Jiang Xu[1], Zi-Yao Fan[1], Kui Xu[1], Nan Wang[1], Yue Wang[1], Jing-Jing Che[1], Zhi-Guo Liu[1], Yu-Lian Mu[1,*], Kui Li[1,2,*]

[1]State Key Laboratory of Animal Nutrition and Key Laboratory of Animal Genetics, Breeding and Reproduction of Ministry of Agriculture and Rural Affairs of China, Institute of Animal Sciences, Chinese Academy of Agricultural Sciences, Beijing, China.

[2]Shenzhen Branch, Guangdong Laboratory of Lingnan Modern Agriculture, Genome Analysis Laboratory of the Ministry of Agriculture and Rural Affairs, Agricultural Genomics Institute at Shenzhen, Chinese Academy of Agricultural Sciences, Shenzhen, China.

[3]College of Animal Science and Technology, Nanjing Agricultural University, Nanjing 210095, China.

[4]Animal Husbandry and Veterinary Department, Beijing Vocational College of Agriculture, Beijing 102442, People's Republic of China

[†]Song-Song Xu, Xiu-Ling Zhang and Sha-Sha Liu contributed equally to this work.

*Corresponding authors: Kui Li, likui@caas.cn; Yu-Lian Mu, mouyulian@caas.cn

RESULTS

Metagenome and Metatranscriptome Sequencing

We respectively sequenced DNA and RNA obtained from the feces and indicated intestinal segment (i.e., ileum, cecum, and colon) contents. A total of ~2.2 Tb and ~700 Gb sequences were generated from gut microbial metagenome and metatranscriptome sequencing (Tables S3-4), respectively. Following quality control and *de novo* assembly, a total of 4,612,189 and 992,787 non-redundant genes were included in the gut microbial metagenomic and metatranscriptomic datasets (Figure 1), respectively.

A Comparison of the Inbred Wuzhishan Minipig Fecal Metagenome with Human and Mouse

Here, we compared our inbred Wuzhishan minipig fecal microbial catalog with two previously published fecal microbial (from human and mouse) gene catalogs (1, 2), both of which were generated using similar (Illumina sequencing) data with similar computational procedures. A much larger number of the minipig fecal bacterial genes mapped to the human catalog as compared to mouse: 35.02% (520247/1485440) of minipig sample bacterial genes were included in the human gut bacterial gene catalogs, compared to only 29% (745908/2572074) of mouse sample bacterial genes. Moreover, only 15.49% (230141/1485440) of minipig bacterial genes were found in the mouse gut bacterial gene catalog.

Moreover, we also compared the microbiome using KO (KEGG orthology) and genus level relative abundances, respectively, to quantify the overlap of the minipig and mouse fecal microbiomes with that of human. Of note, the similarity of annotated KOs among the minipig, human and mouse gut microbiota was very high (minipig and human: Spearman's r = 0.801, P < 0.001; mouse and human: Spearman's r = 0.865, P < 0.001; minipig and mouse: Spearman's r = 0.779, P < 0.001; Figure 2A). We further identified 872 KOs (relative abundance > 0.01%) involved in metabolic functions (e.g., carbohydrate, amino acid, nucleotide, cofactors and vitamins, energy metabolism and glycan biosynthesis), genetic information processing (e.g., translation and replication) and cellular processes (e.g., cell motility) that are shared among the minipig, human and mouse gut microbiomes. However, correlations in abundance for genera showed that the minipig gut microbiome was closer to the human microbiome than the mouse microbiome (minipig and human: Spearman's r = 0.767, P < 0.001; mouse and human: Spearman's r = 0.682, P < 0.001; minipig and mouse: Spearman's r = 0.708, P < 0.001; Figure 2A). We also found bacterial genera that occurred in all samples from minipig, human and mouse. Among the 35 most abundant genera in each species, 14 genera shared were commonly detected as gut microbiota (Figure 2B), including previously reported genera like *Prevotella*, *Bacteroides*, *Clostridium*, *Eubacterium*, *Parabacteroides* and *Ruminococcus* and so on (3). Taken together, the minipig gut microbiome had a higher taxonomic and functional overlapped with the human gut microbiome.

RNA Sequencing and Identification of LncRNA and mRNA

RNA-seq-based transcriptome profilings revealed a total of 1,727 million raw reads and 1,638 million clean reads after quality control (Table S10). The percentage of clean reads compared to raw reads for each library ranged from 90.03% to 96.96%. Among the clean reads, the percentage of reads with Q30 ranged from 87.30% to 93.17%, and the average GC content was 49.87%. The clean reads of all samples were then considered for alignment with *Sus scrofa* genome (https://www.ncbi.nlm.nih.gov/genome/?term=pig). The mapped rates of 16 samples ranged from 71.91% to 77.91% (Table S10). Thus, these results suggested that the RNA-seq data was stable and reliable. We totally identified 25,491 mRNAs from both the HED and ND groups.

Figure Legends

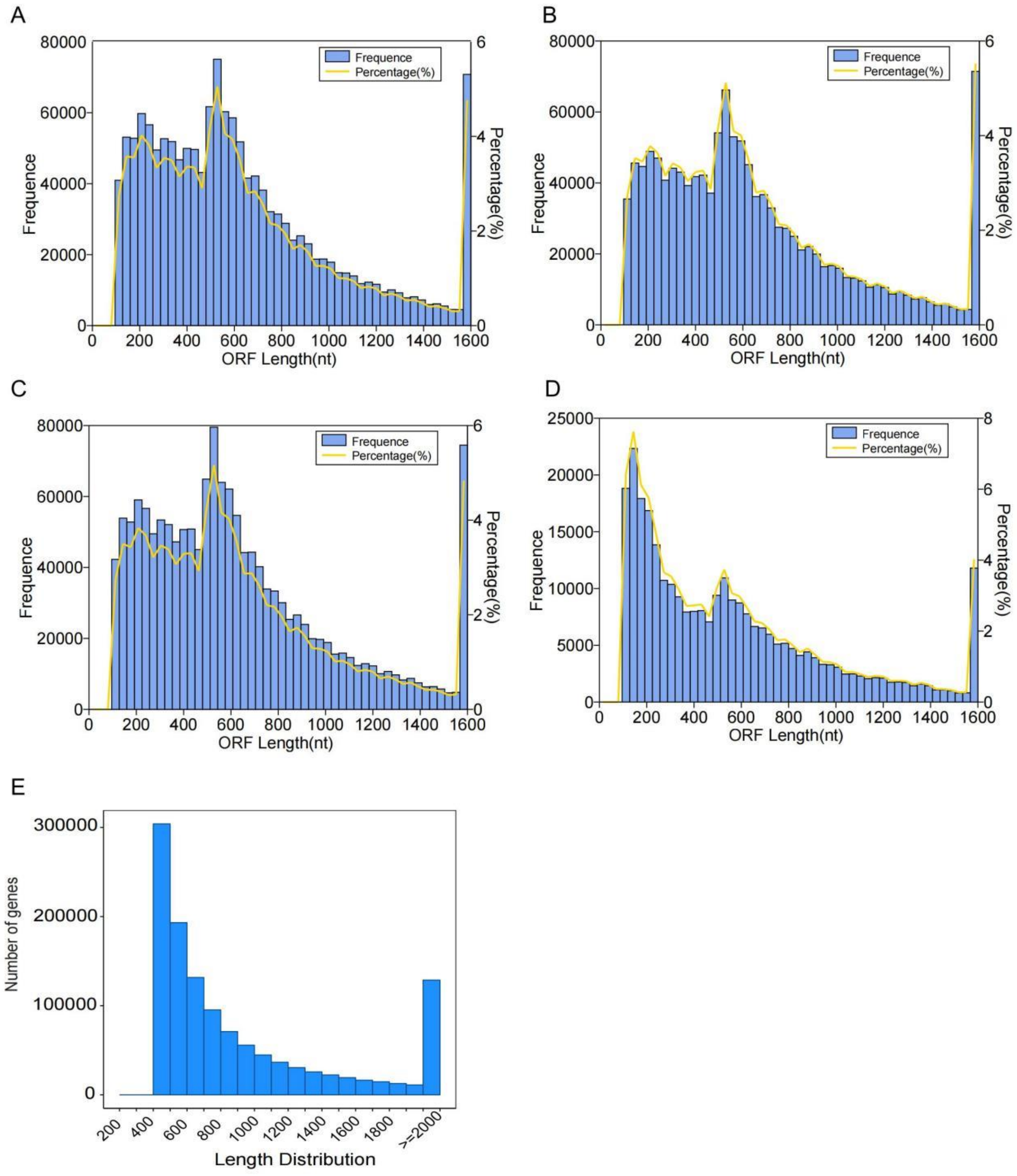

FIGURE 1 The length distribution of non-redundant genes for (**A**) fecal, (**B**) cecal, (**C**) colonic and (**D**) ileal microbiota in metagenome dataset; (**E**) Feces and the indicated intestinal segment contents in metatranscriptome dataset.

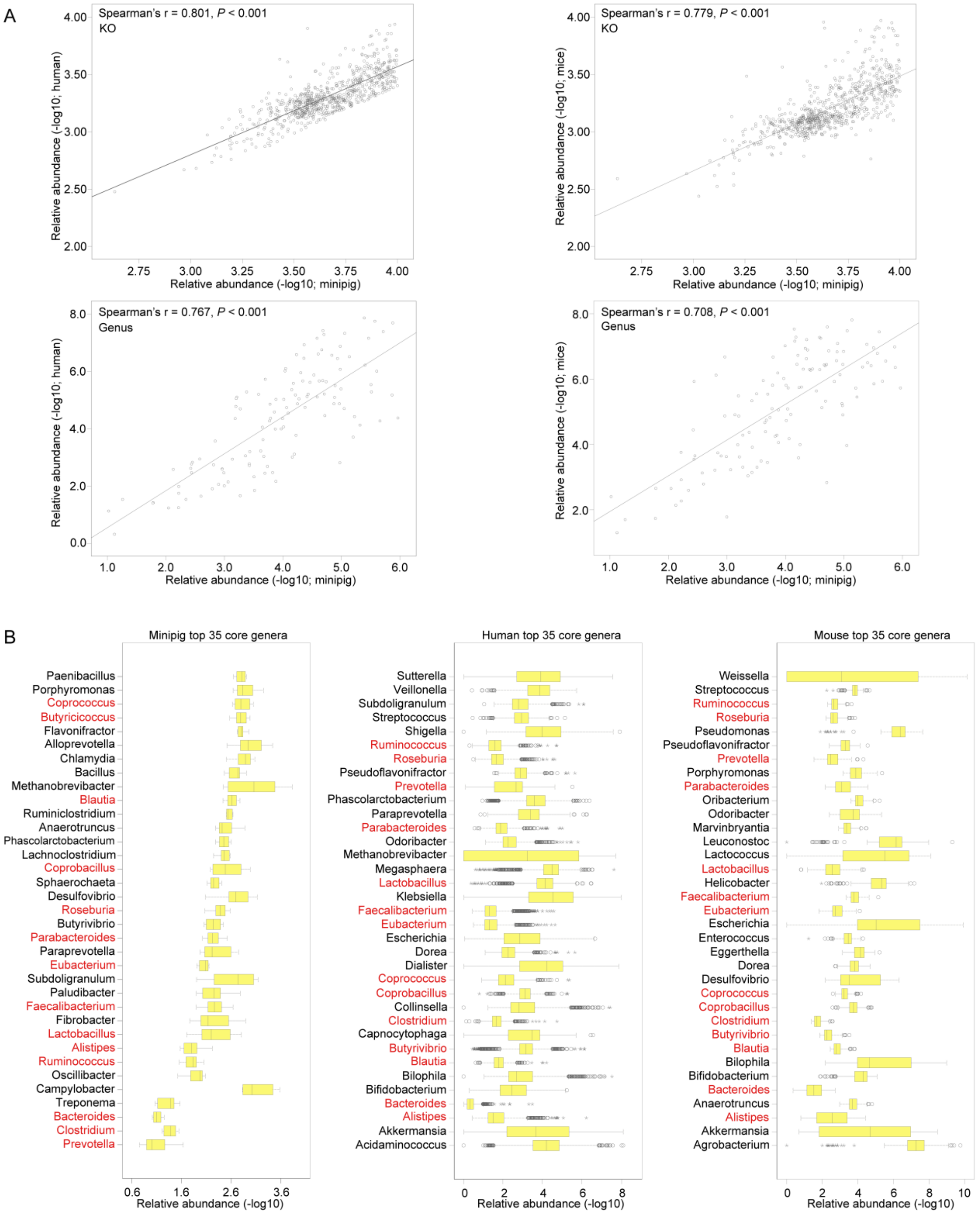

FIGURE 2 Comparisons of the inbred minipig, human and mouse fecal microbiomes. (**A**) Spearman correlation between the minipig and human and mouse based on KOs and genus abundance estimates ($-\log_{10}$) of KOs and genera with a relative abundance higher than 0.01%, in at least 50% of individuals. (**B**) The top 35 ranking genera in the minipig, human, and mouse gut microbiota. The shared genera are marked in red.

References

1. Li JH, Jia HJ, Cai XH, Zhong HZ, Feng Q, Sunagawa S, et al. An integrated catalog of reference genes in the human gut microbiome. *Nat Biotechnol.* (2014) 32:834-841. doi: 10.1038/nbt.2942

2. Li X, Liang S, Xia Z, Qu J, Liu H, Liu C, et al. Establishment of a Macaca fascicularis gut microbiome gene catalog and comparison with the human, pig, and mouse gut microbiomes. *Gigascience*. (2018) 7(9): giy100. doi: 10.1093/gigascience/giy100

3. Xiao L, Feng Q, Liang S, Sonne SB, Xia Z, Qiu X, et al. A catalog of the mouse gut metagenome. *Nat Biotechnol*. (2015) 33:1103-1108. doi:10.1038/nbt.3353
