# Supplementary figures and images for: Multi-Omic Analysis in a Metabolic Syndrome Porcine Model Implicates Arachidonic Acid Metabolism Disorder as a Risk Factor for Atherosclerosis
Source: Front Nutr. 2022 Feb 23;9:807118. doi: 10.3389/fnut.2022.807118 (PMC8906569; doi:10.3389/fnut.2022.807118)

A

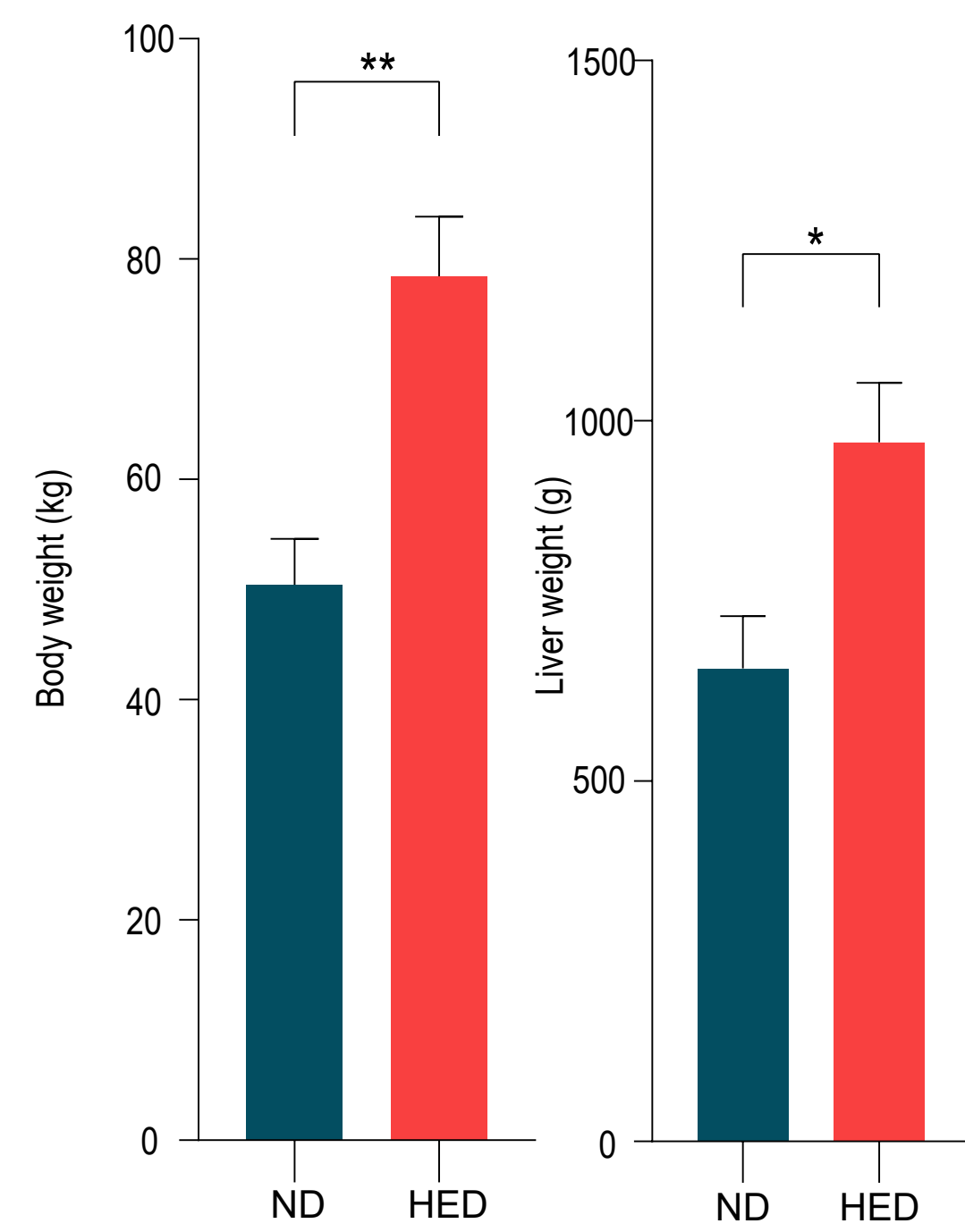

B

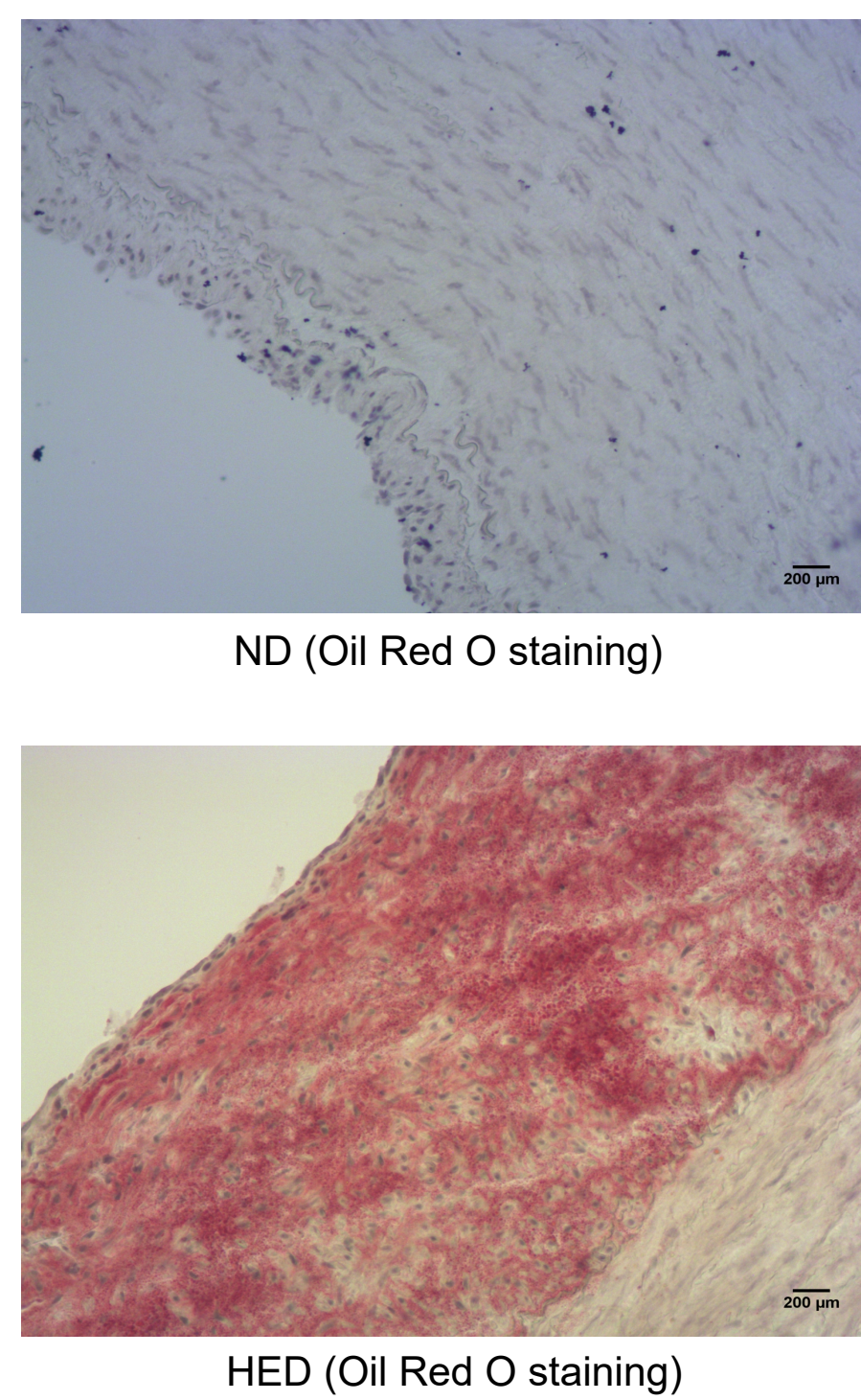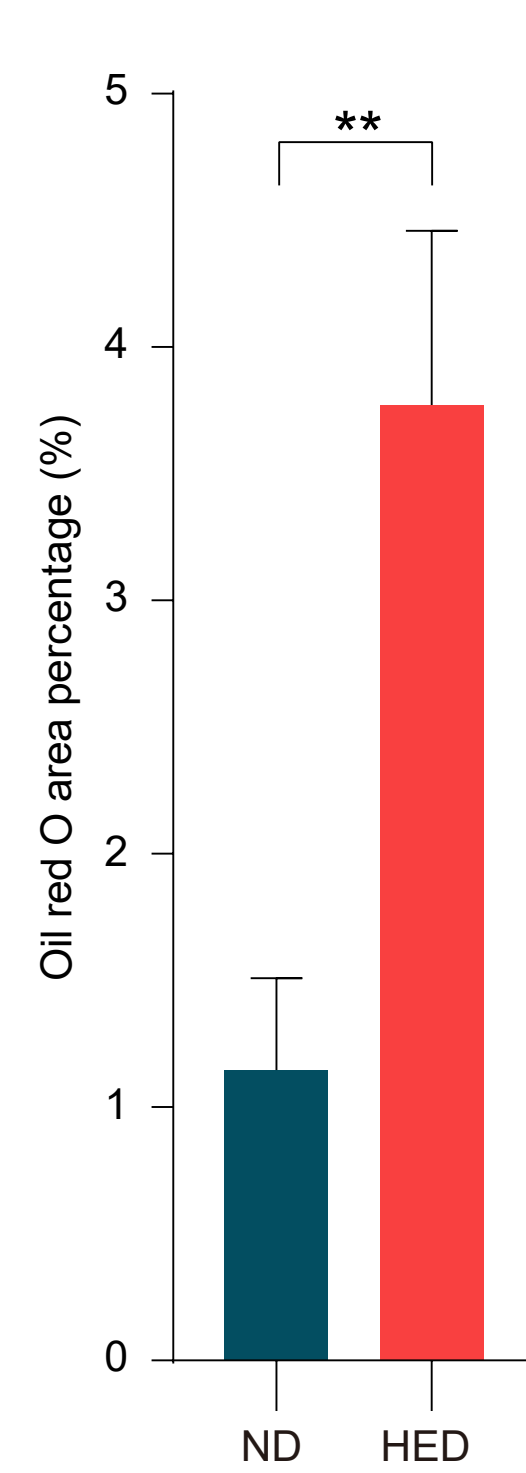

D

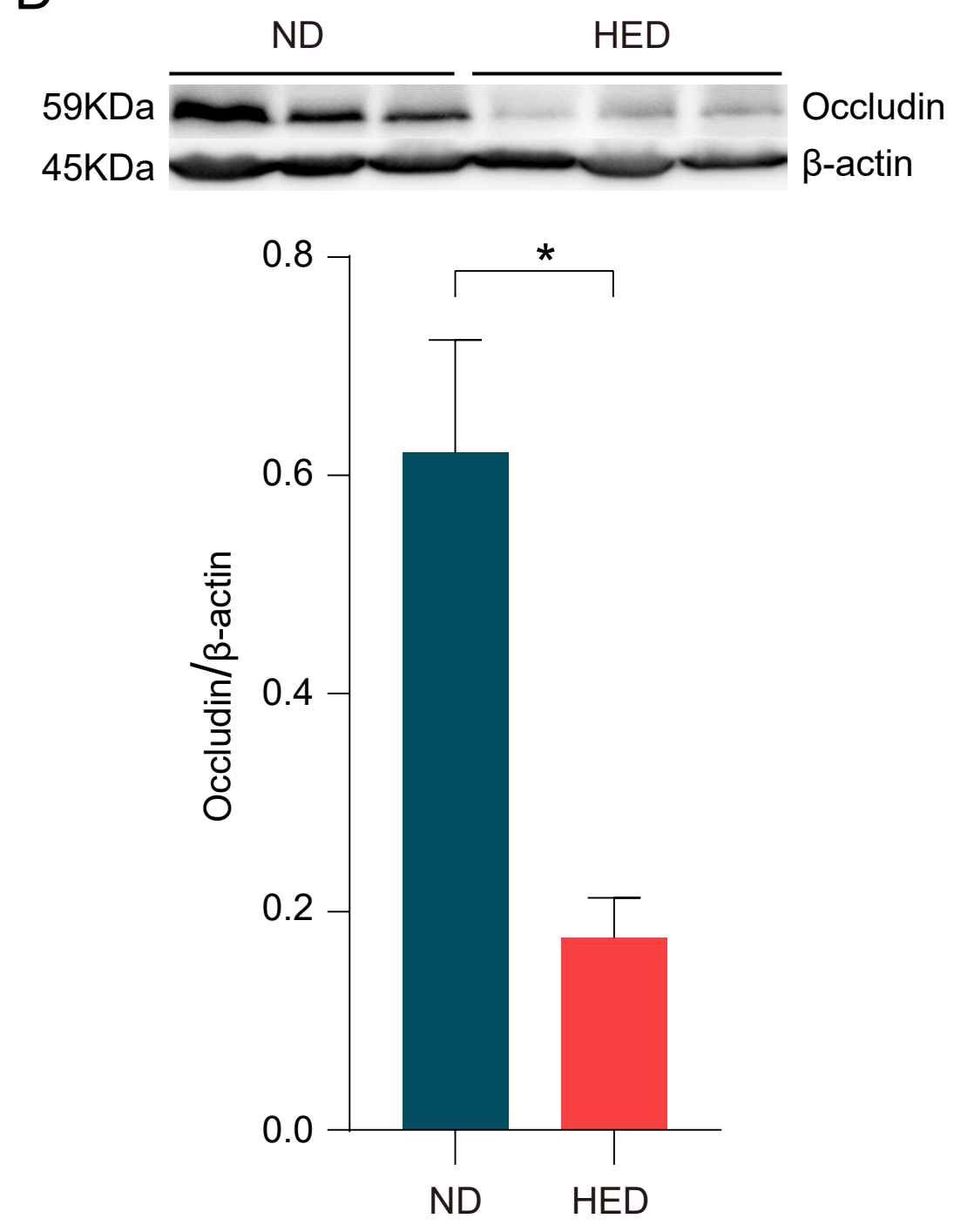

E

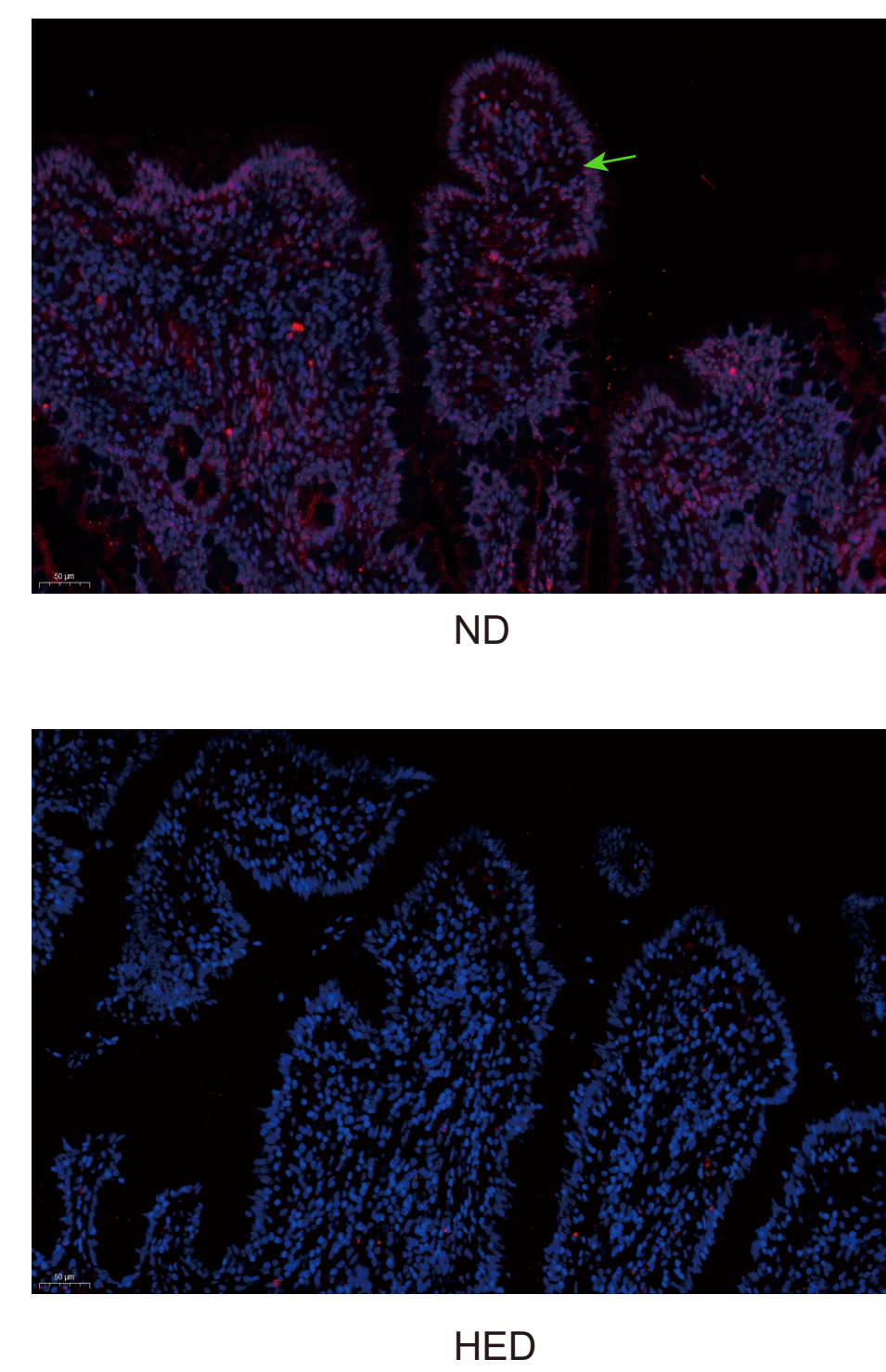

C

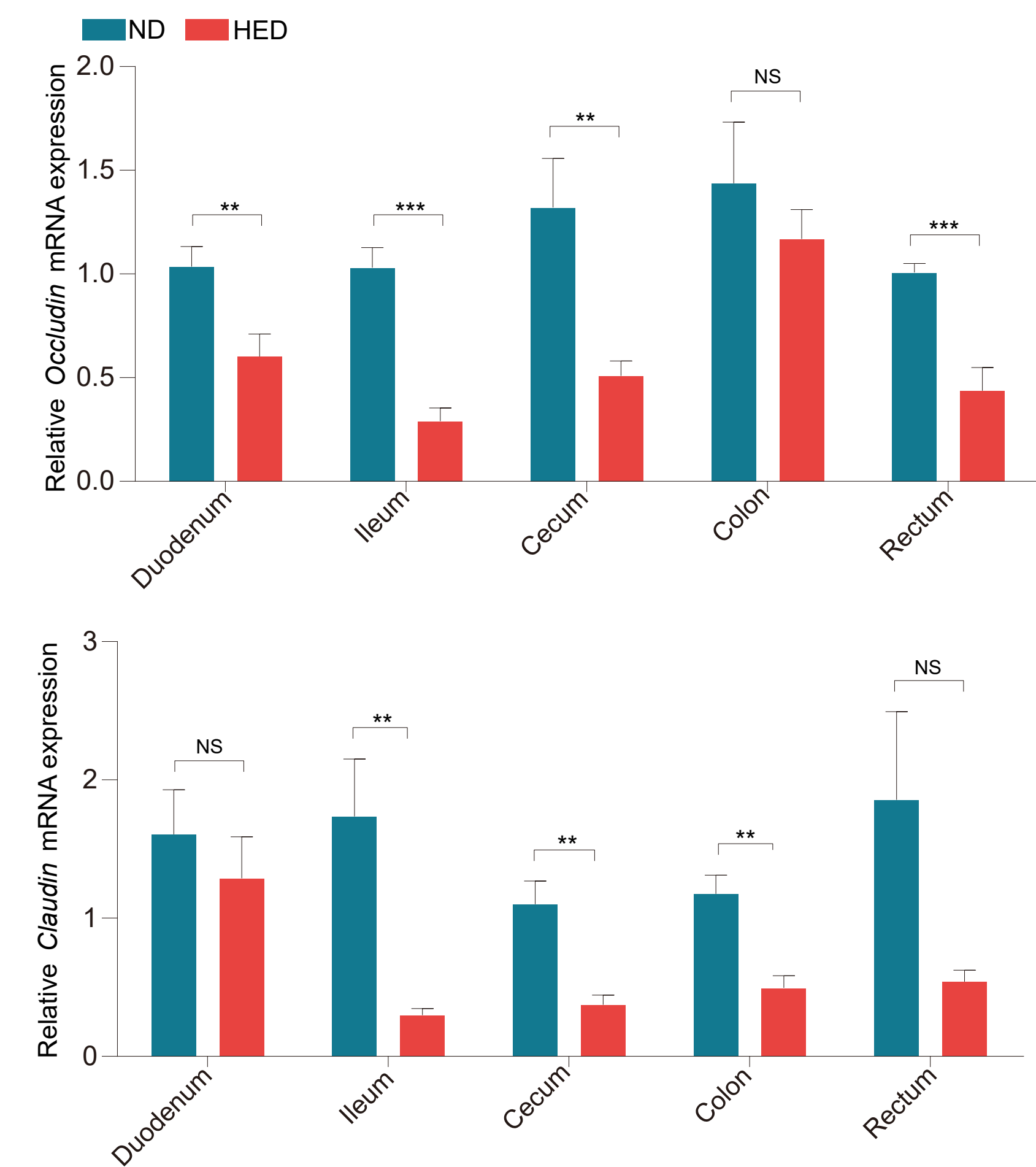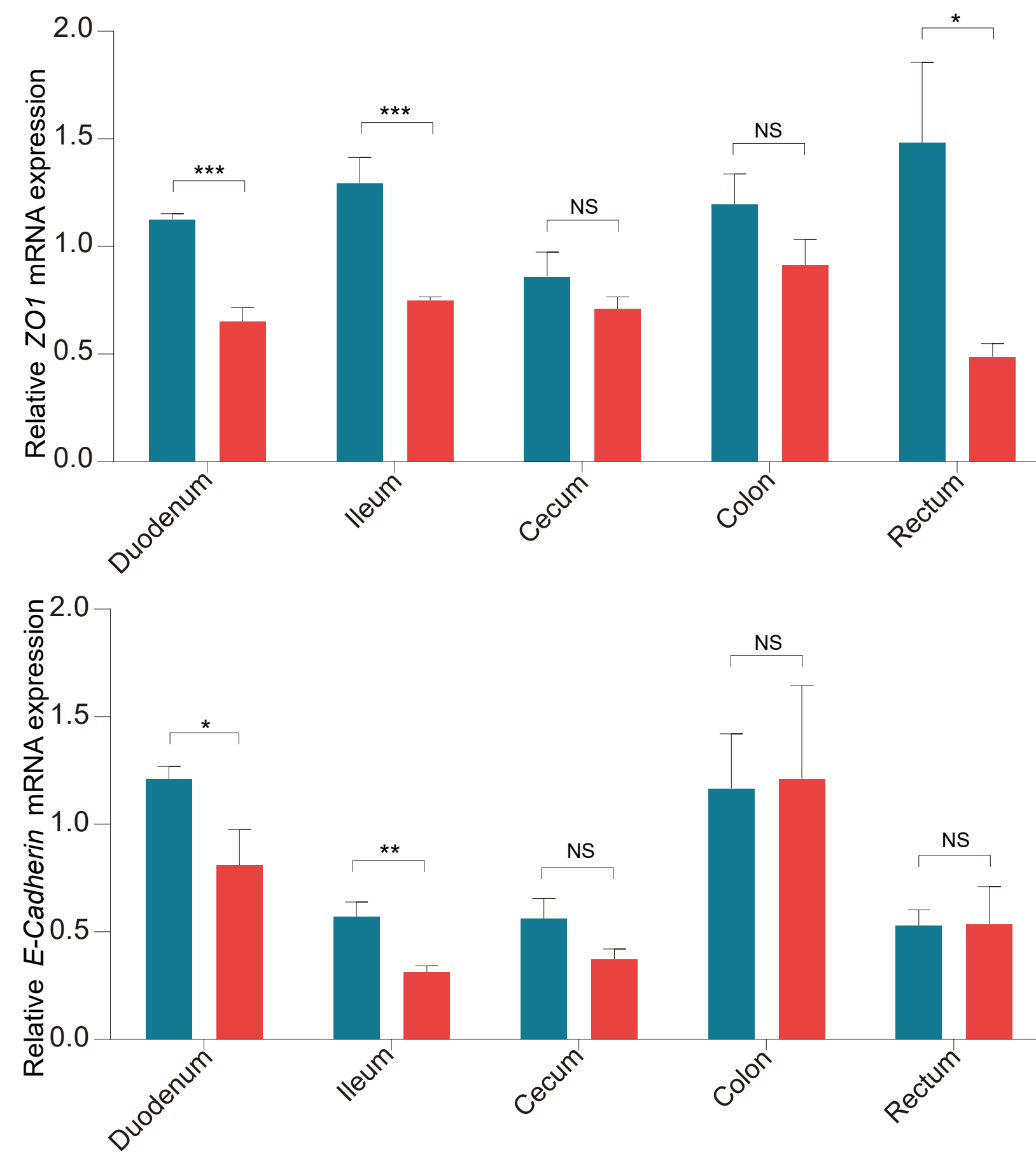

Supplement: Supplementary Figure 1 — (A) Comparison of the body weight and liver weight in the HED and ND groups. (B) Characterization of abdominal aorta cross-sections based on Oil Red O staining (bars, 200 μm). (C) The expression of tight junction genes including Occludin, ZO-1, Claudin, and E-Cadherin in tissues from five intestinal sections (duodenum, ileum, cecum, colon, and rectum) between the HED and ND groups (n = 4 in each group). (D) Intestinal protein expression of Occludin in the HED and ND groups. (E) Immunofluorescence analysis of the Occludin protein in the ileal segment (bars, 50 μm). The red signals are for Occludin; the blue signals are for DAPI (nuclear staining). The arrows pointed at the Occludin protein. Data are shown as the mean ± SEM. *P < 0.05, **P < 0.01, based on student's t-test. [file Data_Sheet_1.PDF]

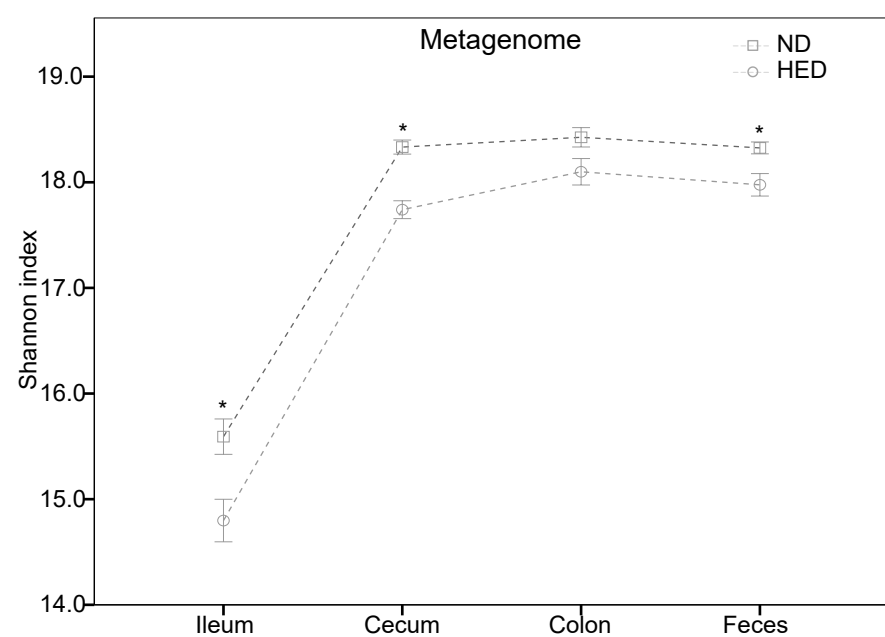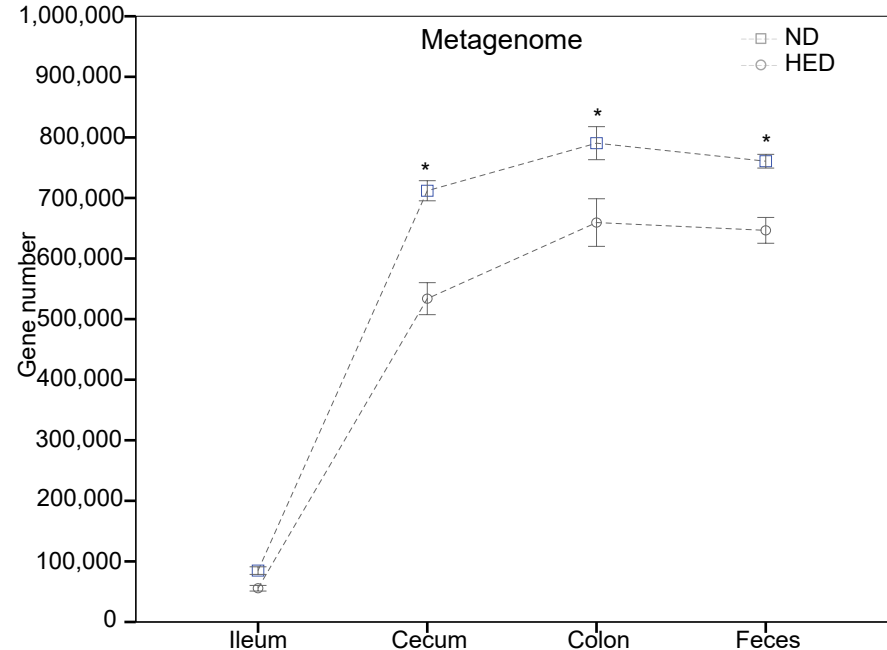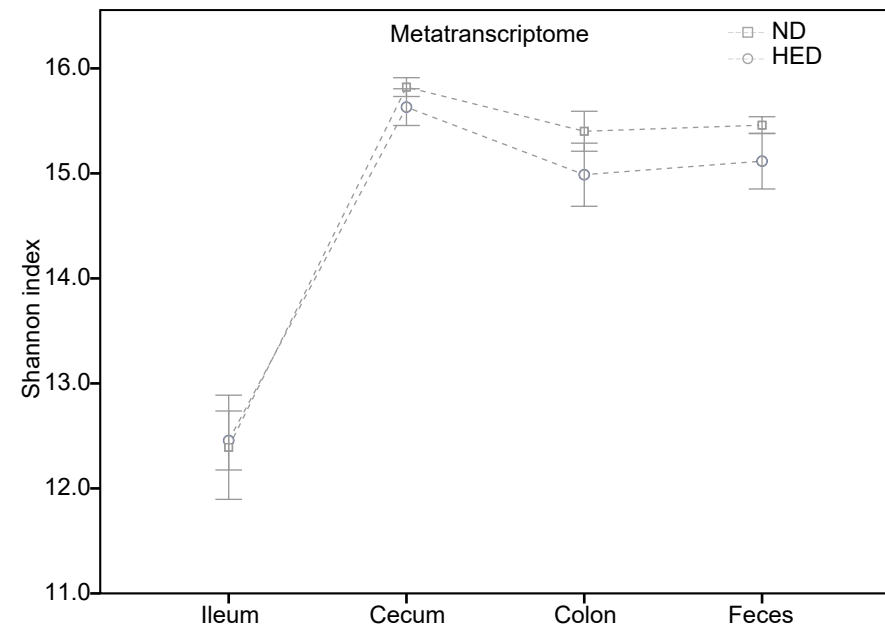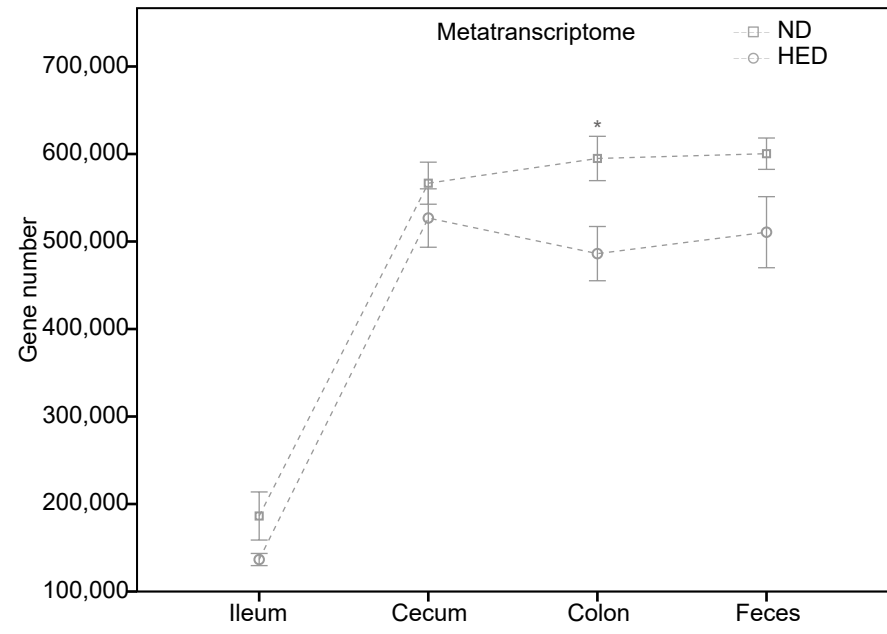

Supplement: Supplementary Figure 2 — Comparison of microbial alpha diversity (Shannon index) and gene richness at the genus level between the HED and ND groups across the indicated intestinal contents and feces. *P < 0.05, based on student's t-test (two-tailed). [file Data_Sheet_2.PDF]

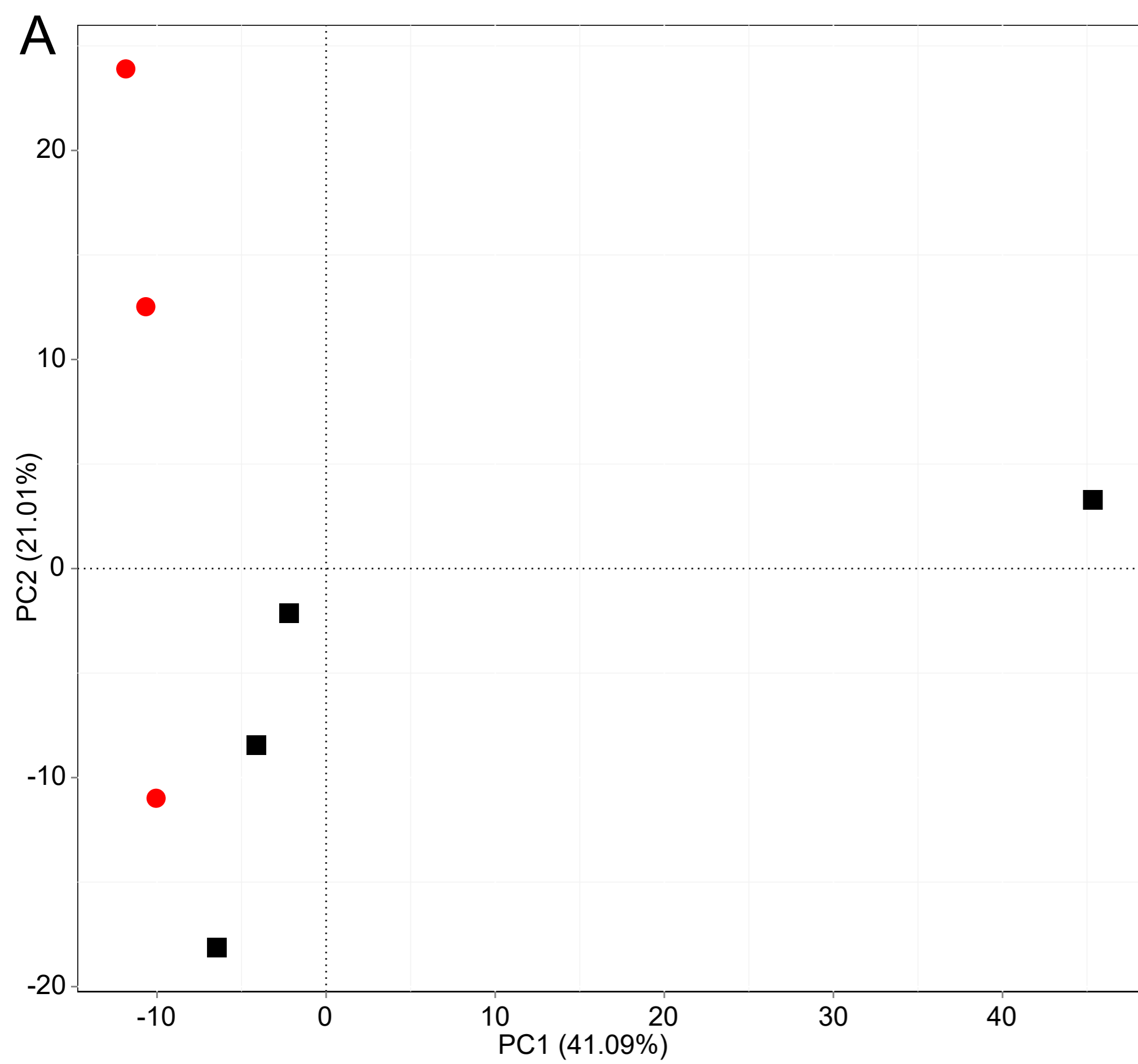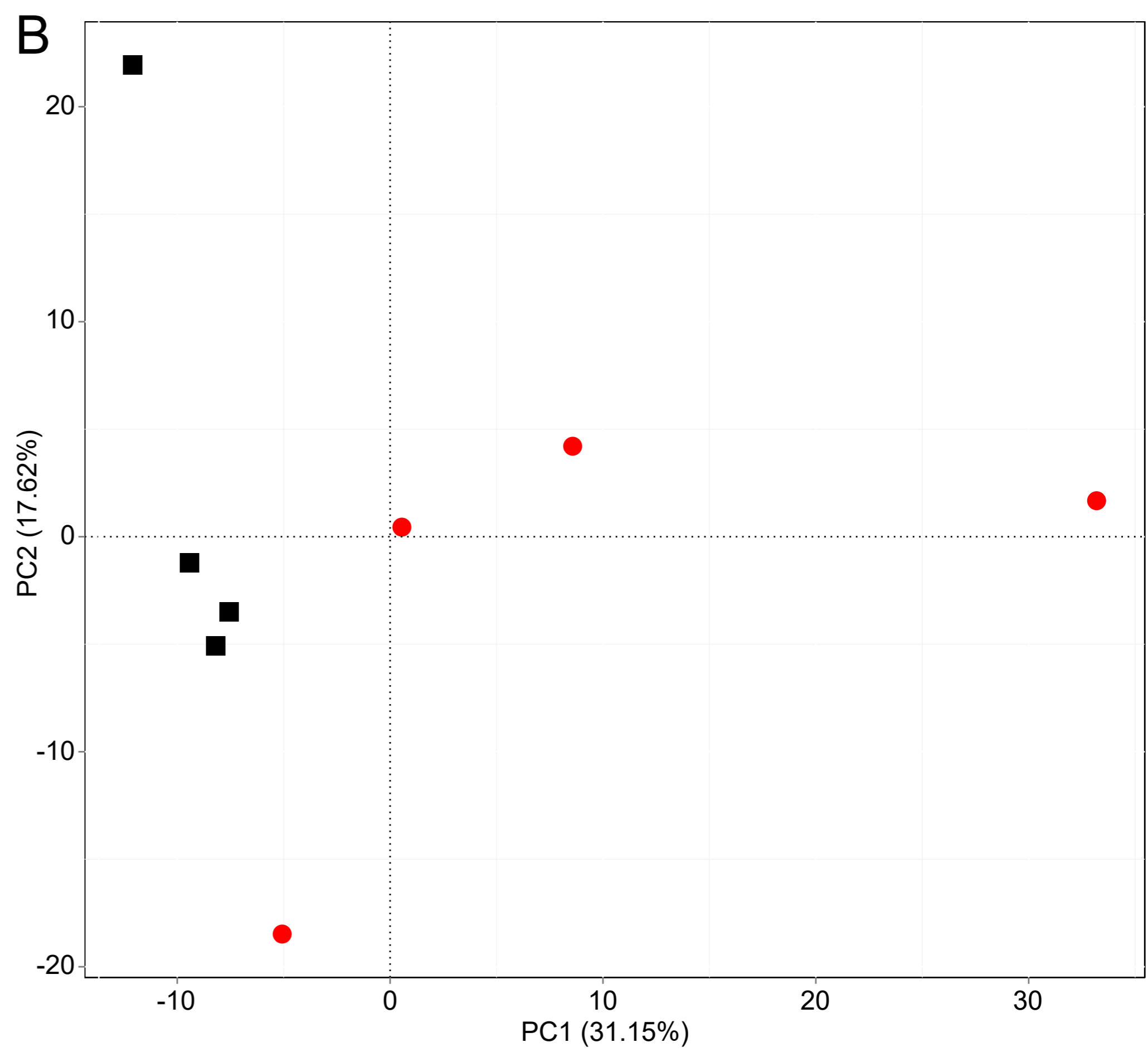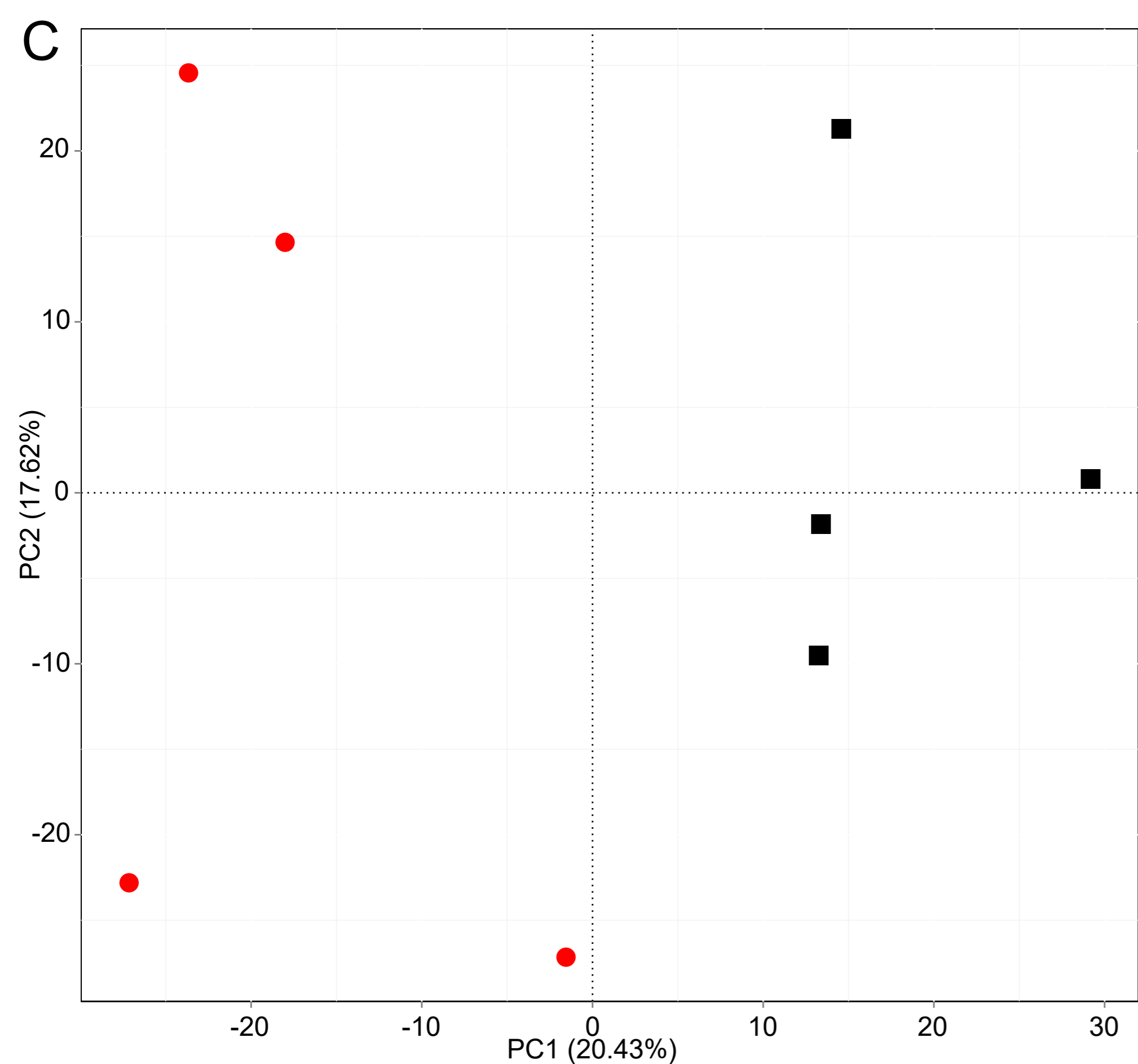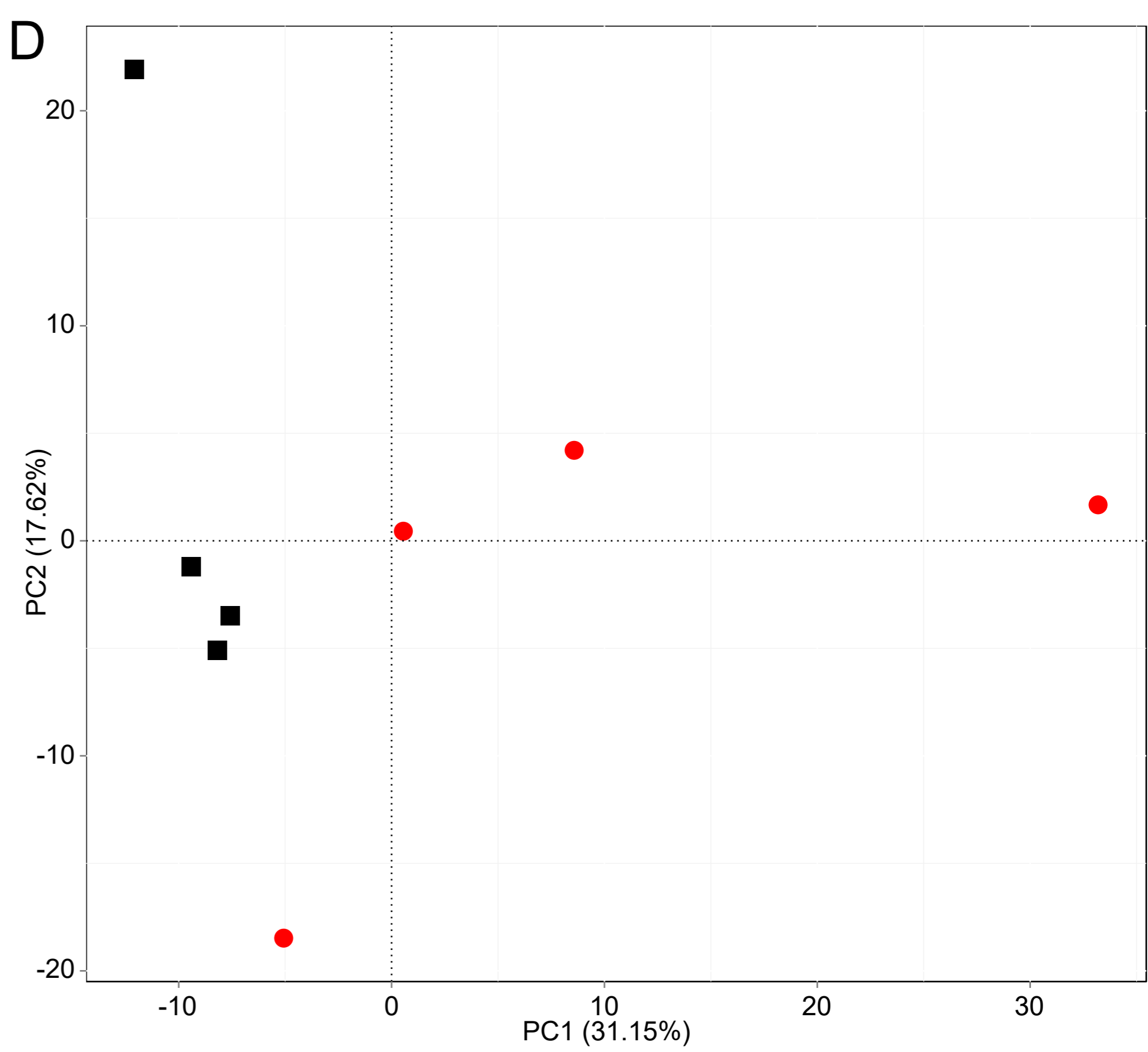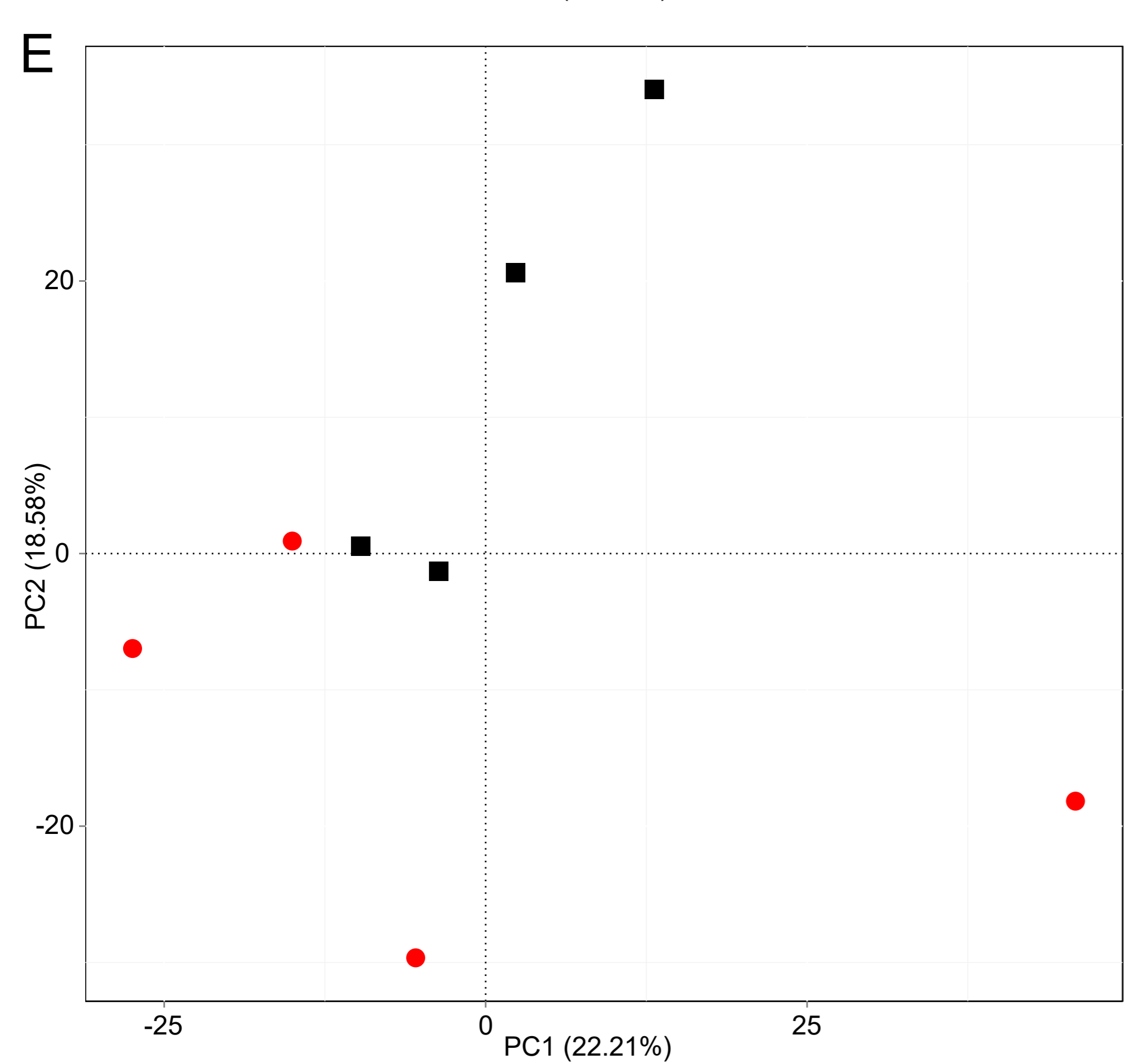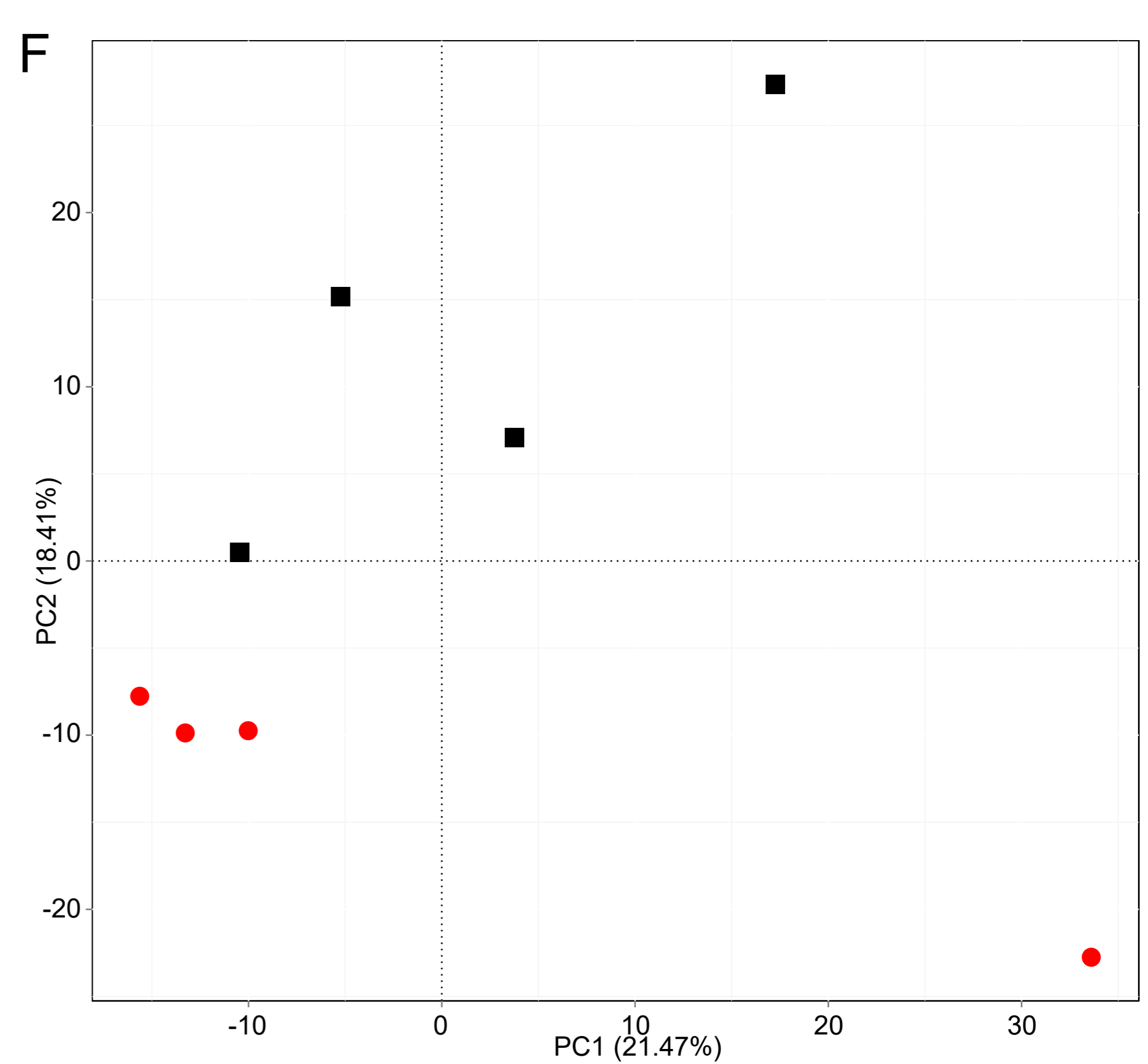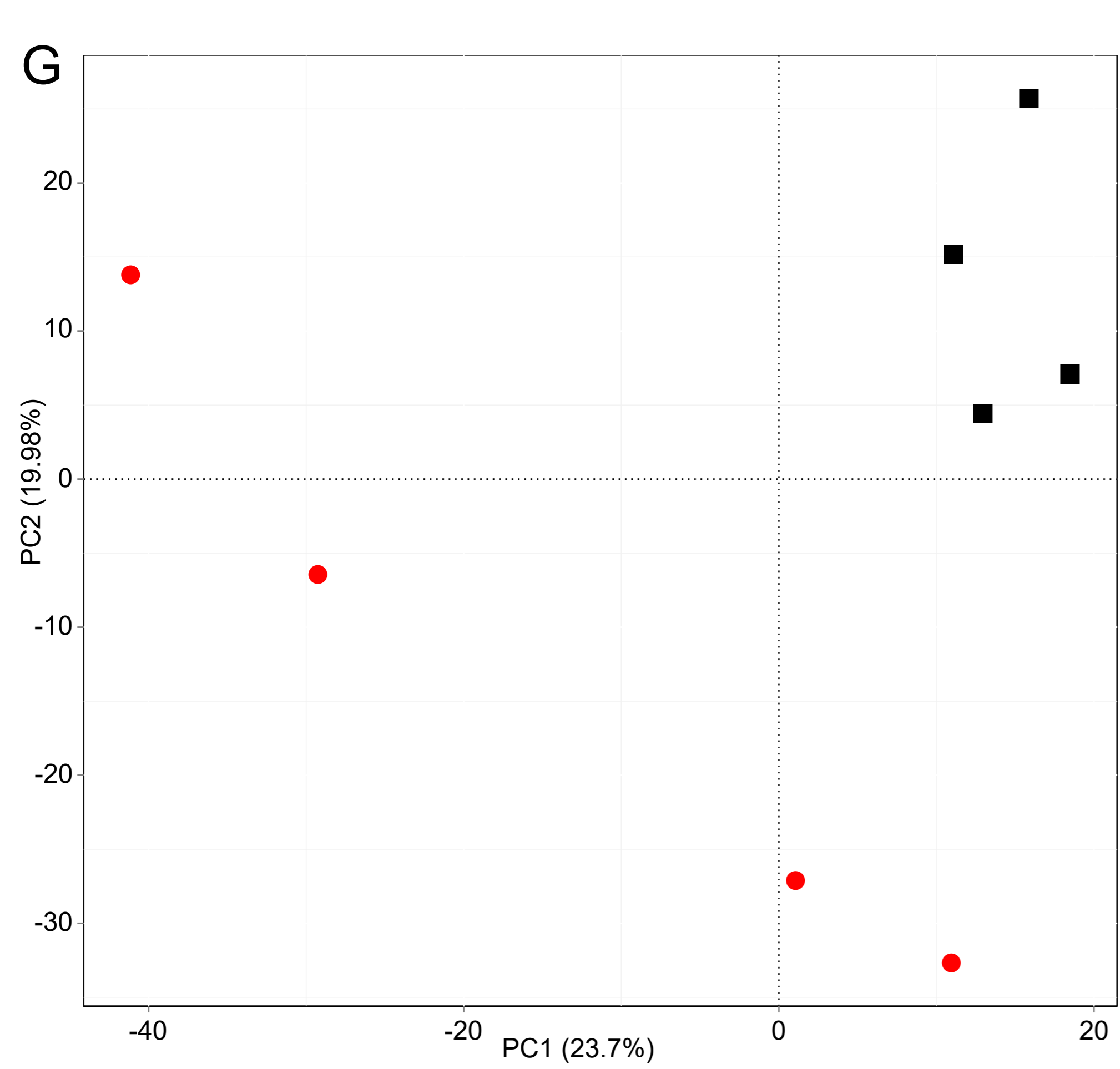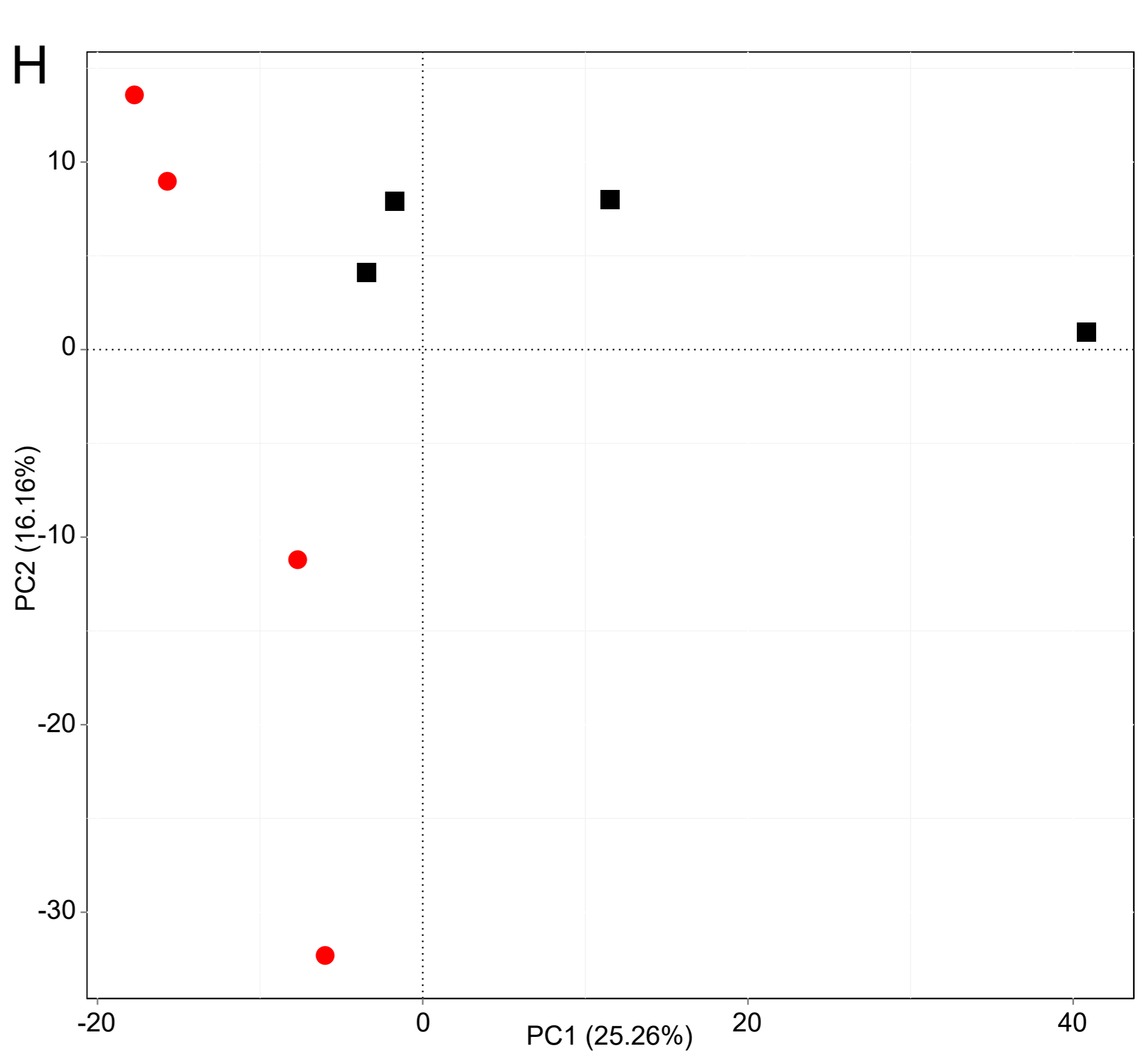

Supplement: Supplementary Figure 3 — Principal component analysis based on genus abundances between the HED and ND groups in the metagenome and metatranscriptome datasets. The (A) metagenome and (B) metatranscriptome datasets of the ileal microbiome. The (C) metagenome and (D) metatranscriptome datasets of the cecal microbiome. The (E) metagenome and (F) metatranscriptome datasets of the colonic microbiome. The (G) metagenome and (H) metatranscriptome datasets of fecal sample microbiome. Red dot represents the HED group animals; black box represents the ND group animals. [file Data_Sheet_3.PDF]

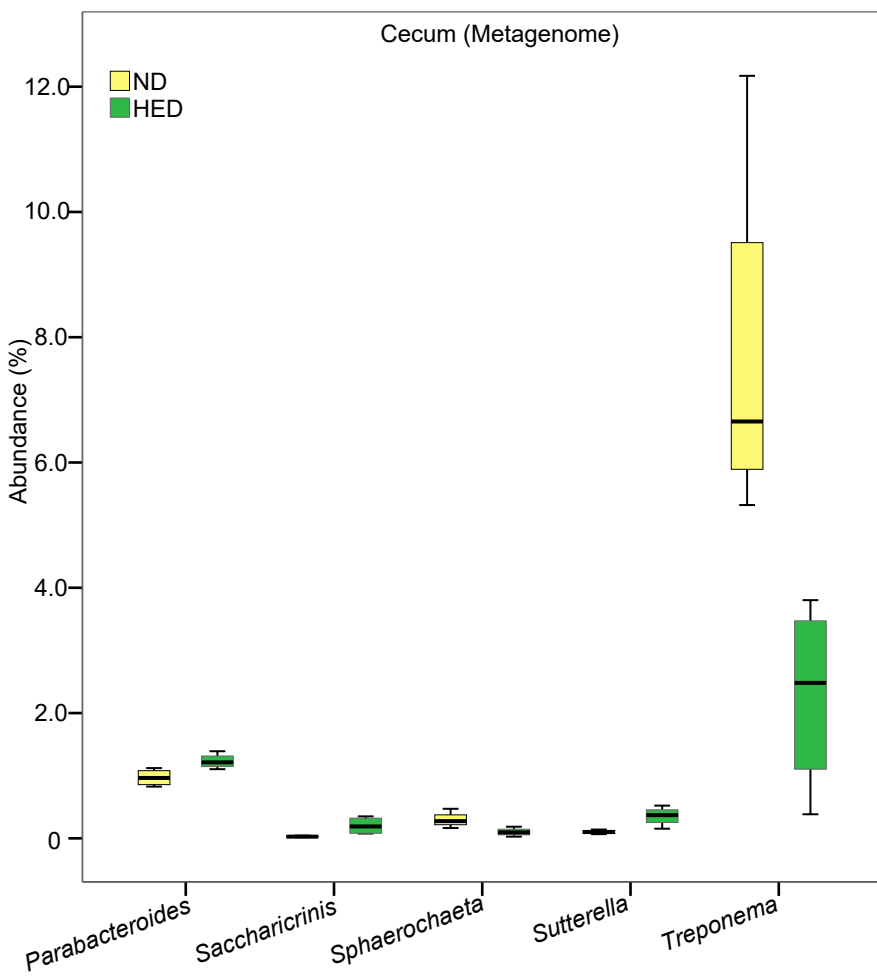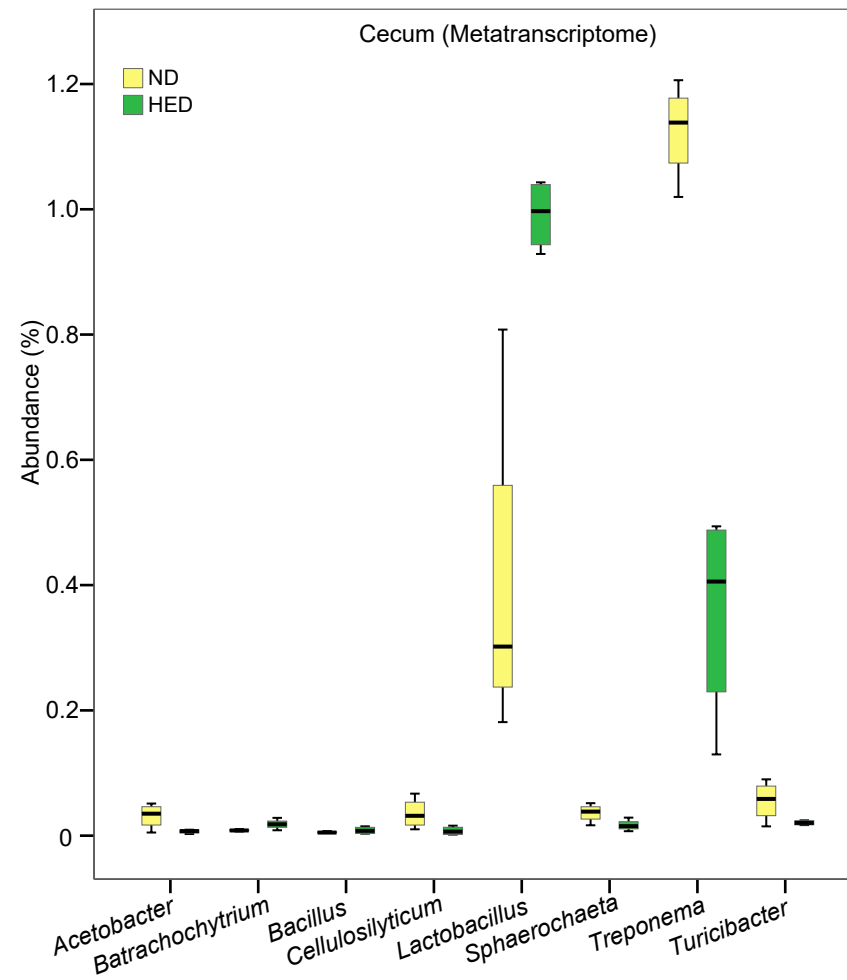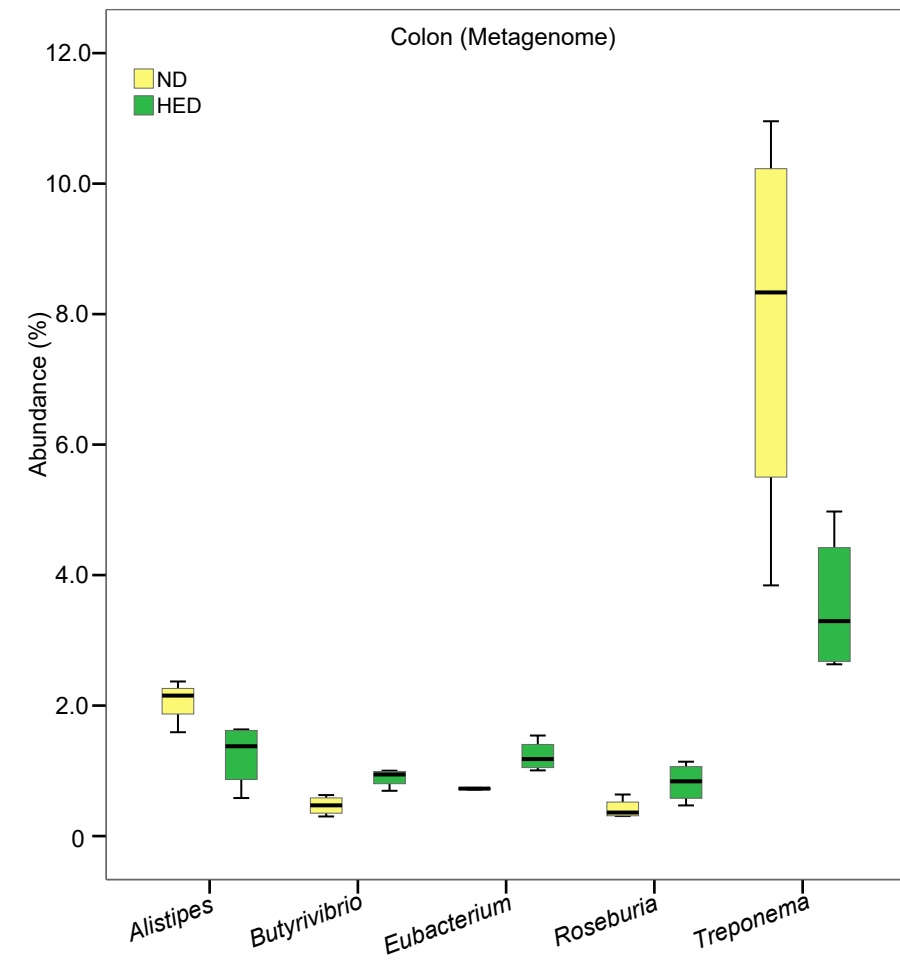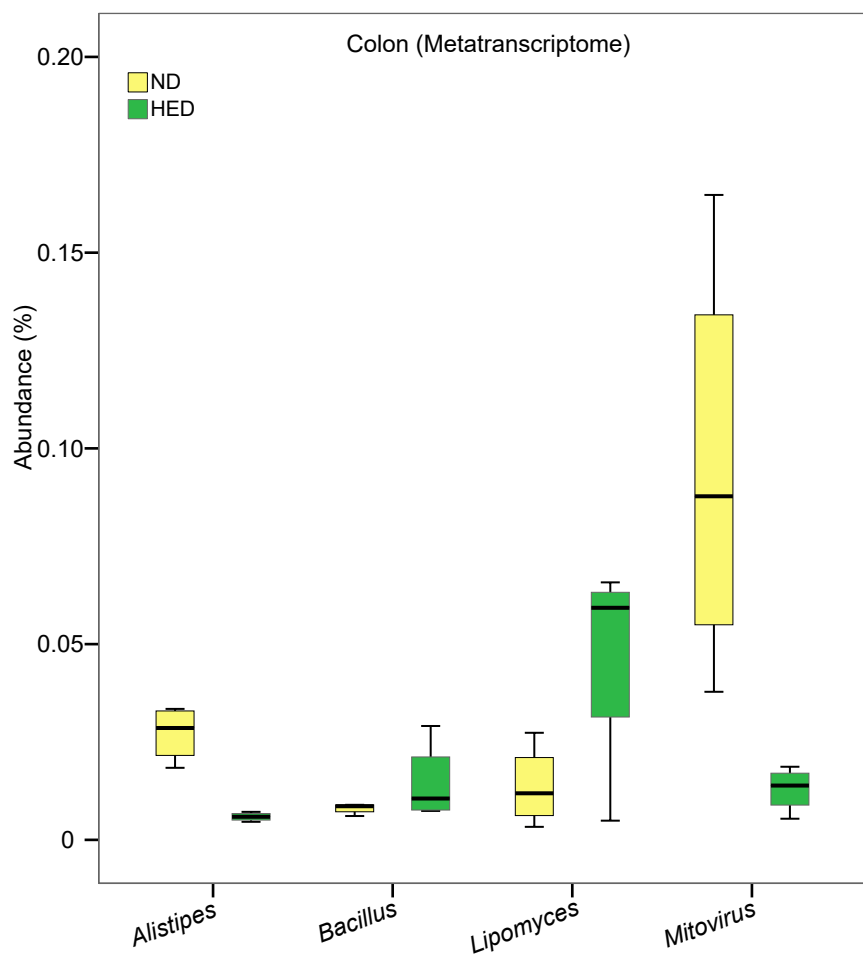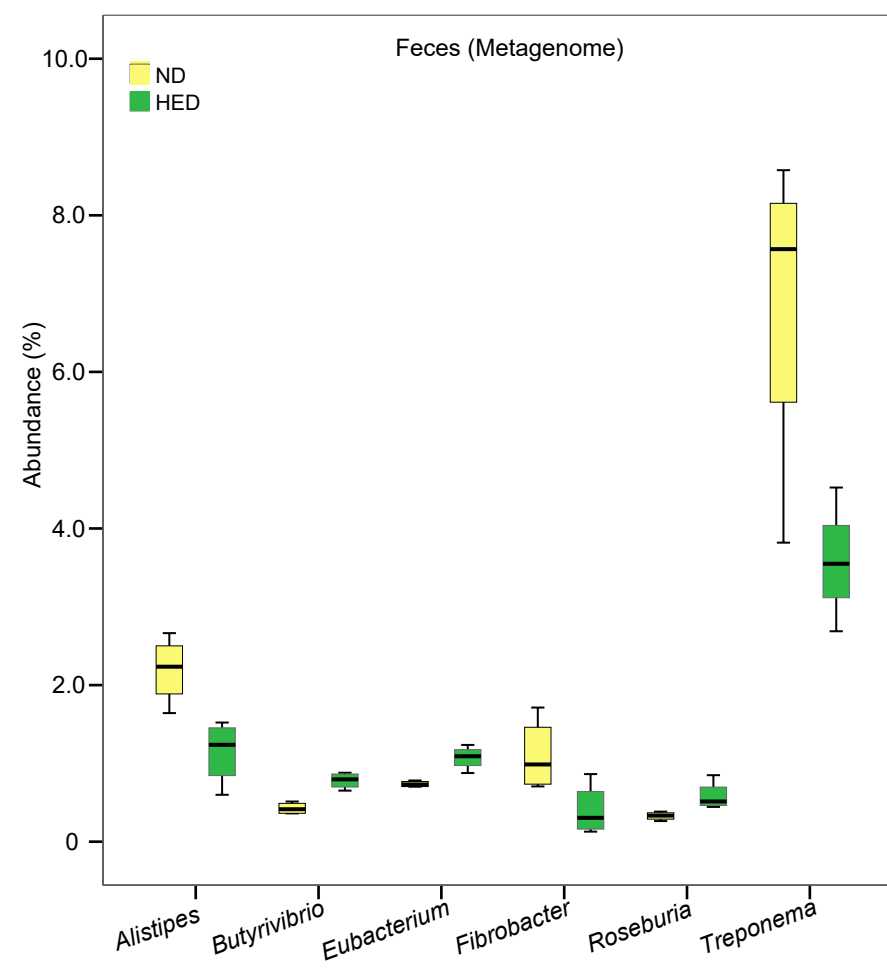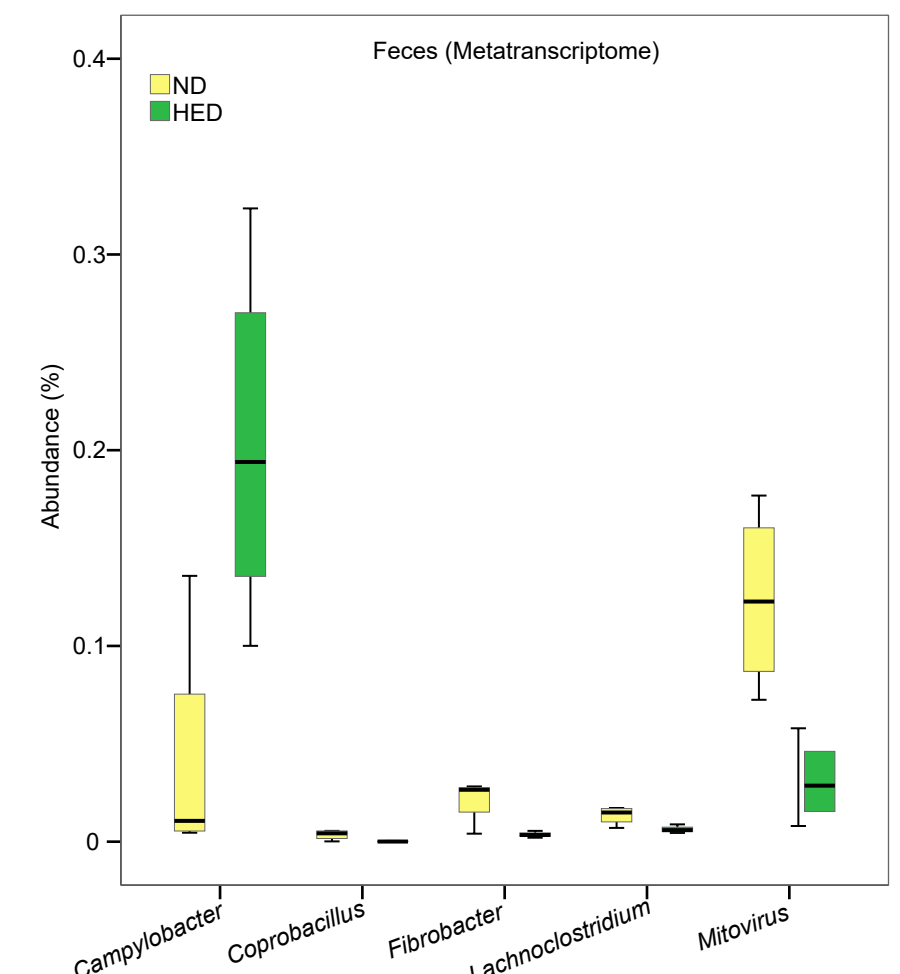

Supplement: Supplementary Figure 4 — Significant differences (P < 0.05; metastats analysis) in the abundances of microbial genera detected between the HED and ND groups based on the metagenome and metatranscriptome datasets in the indicated intestinal contents and feces. [file Data_Sheet_4.PDF]

A

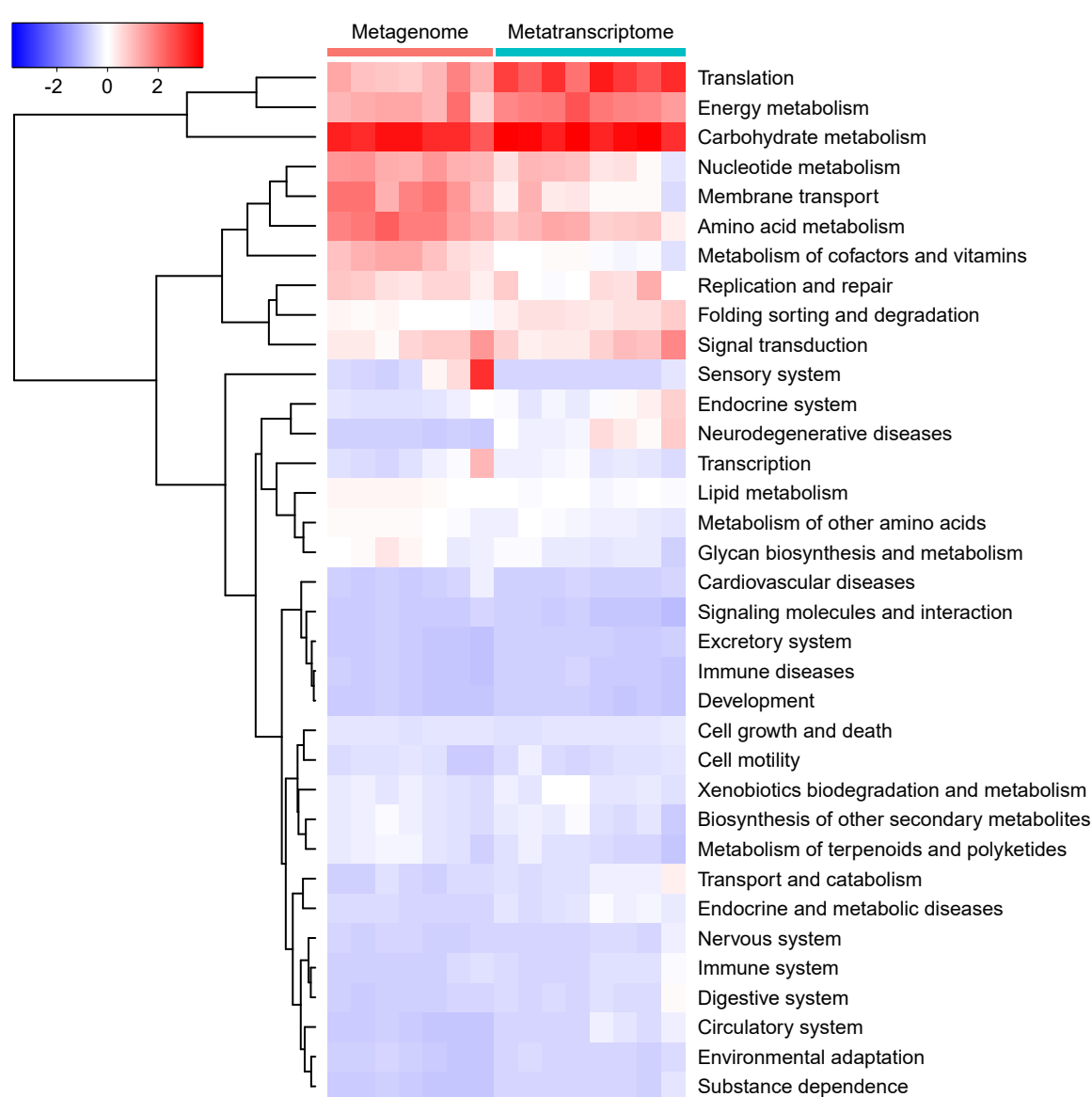

B

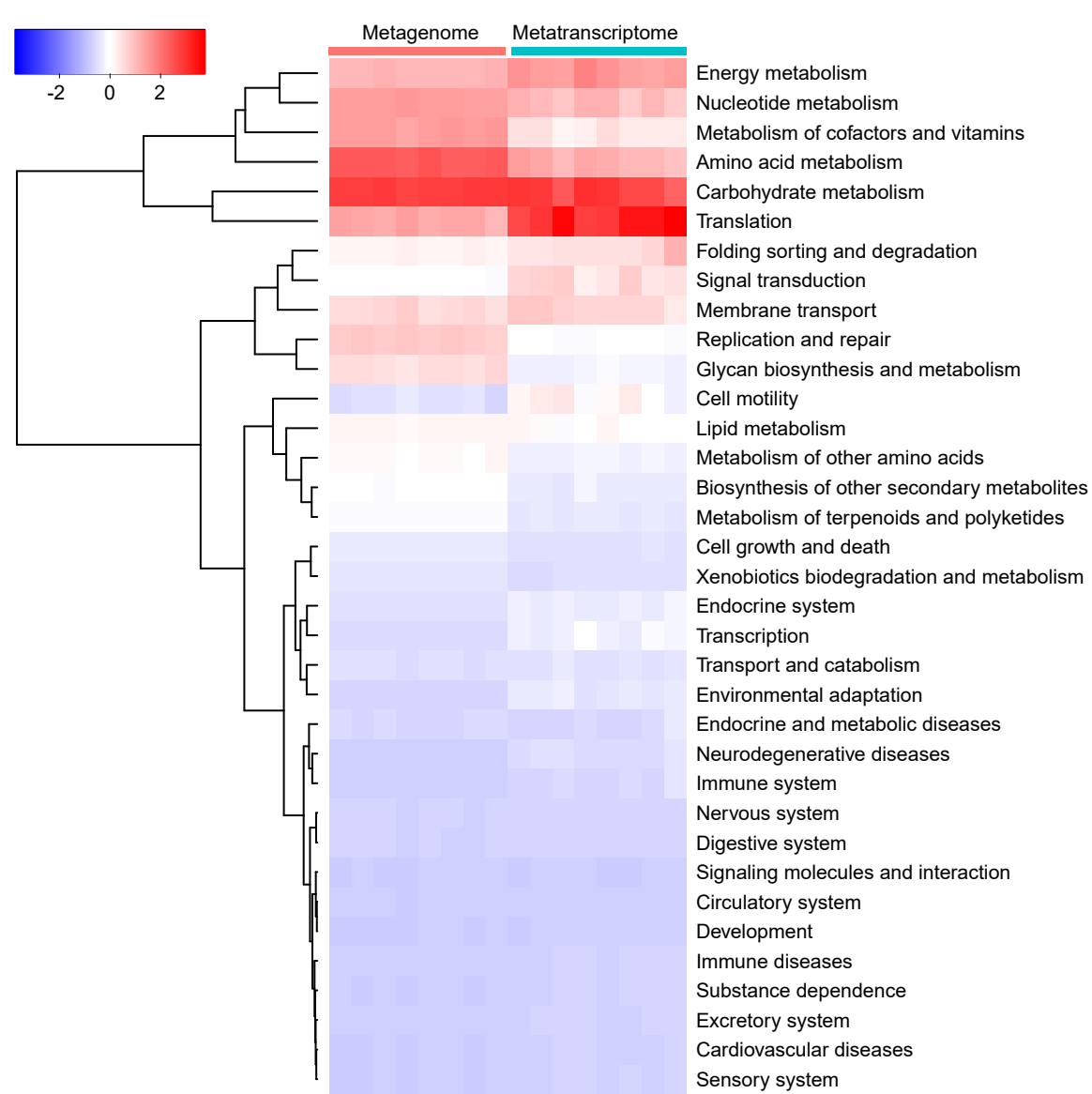

C

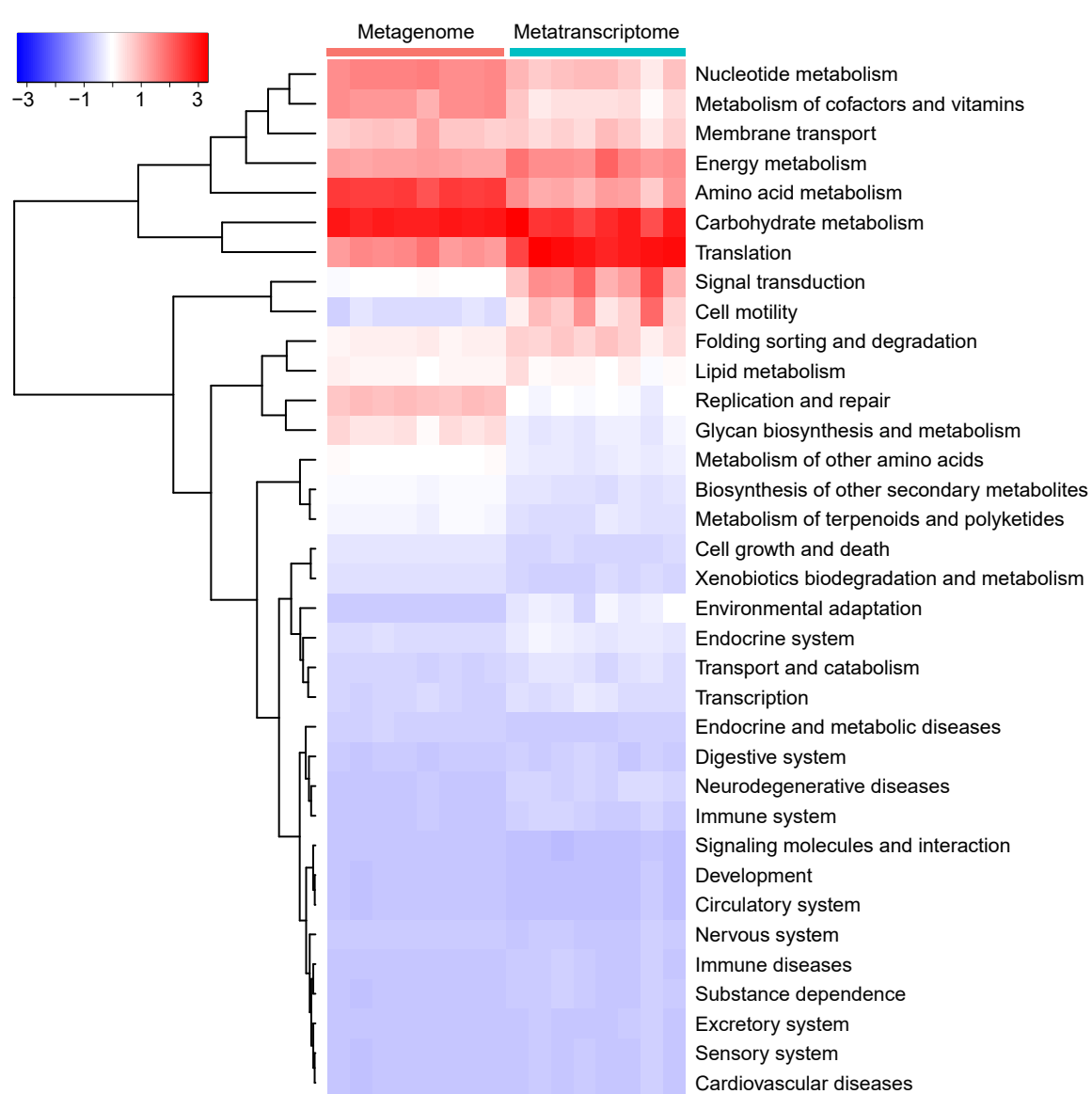

D

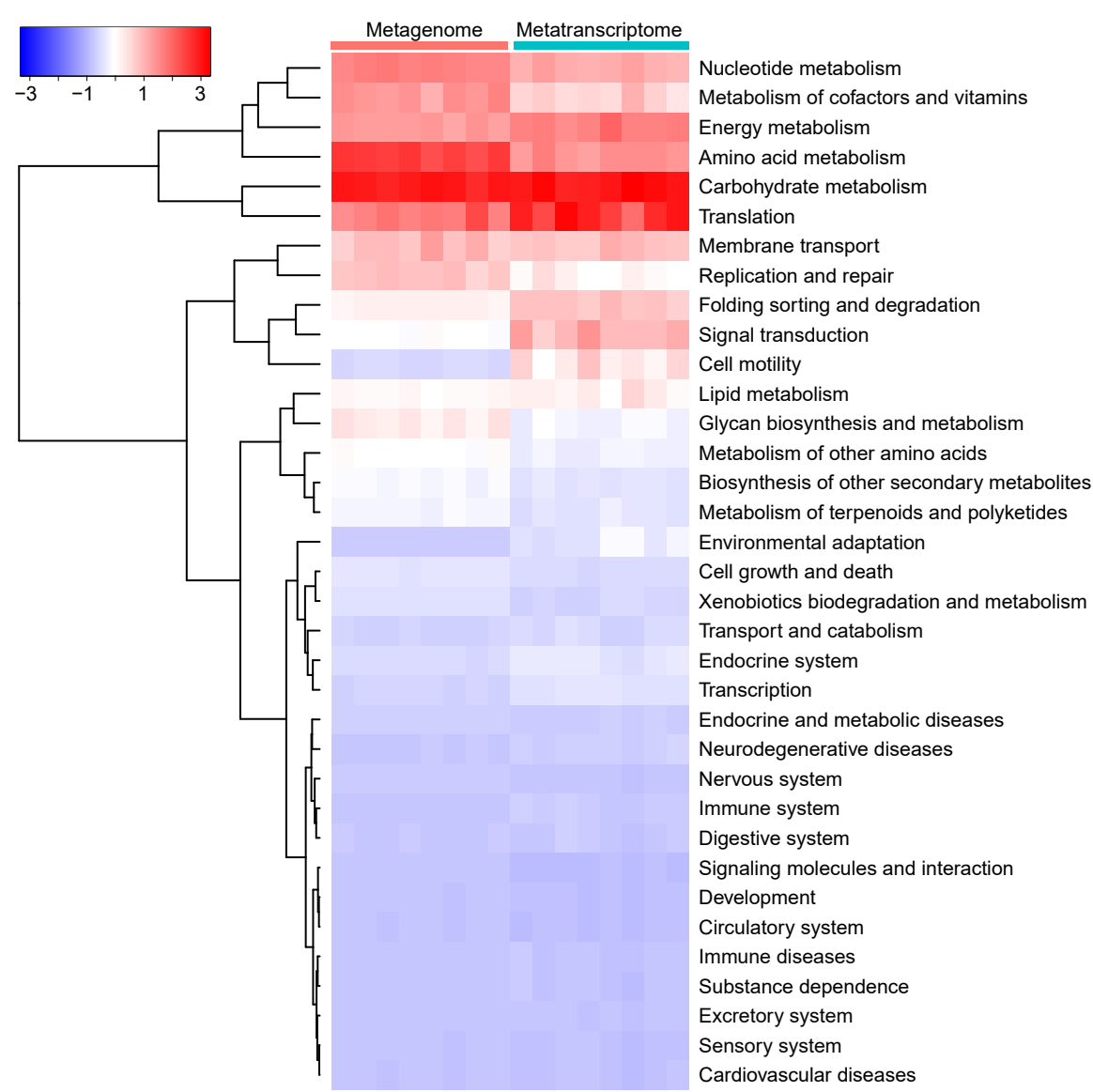

Supplement: Supplementary Figure 5 — KEGG functional categories (level 2) of (A) ileal, (B) cecal, (C) colonic, and (D) fecal microbiome in the metagenome and metatranscriptome datasets. [file Data_Sheet_5.PDF]

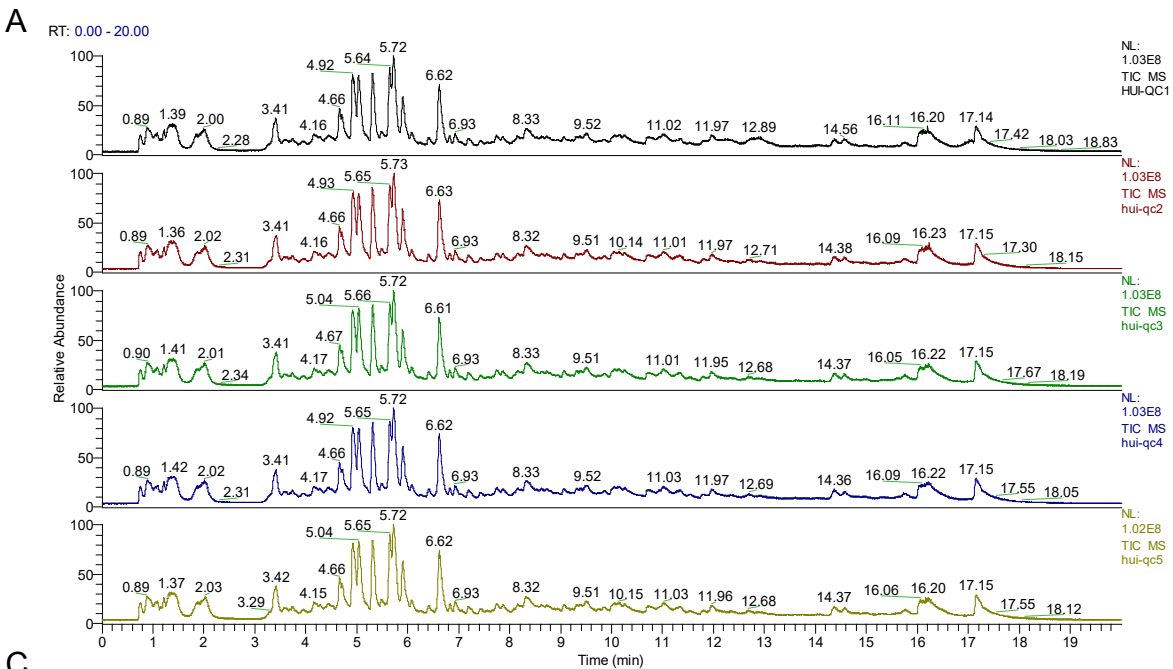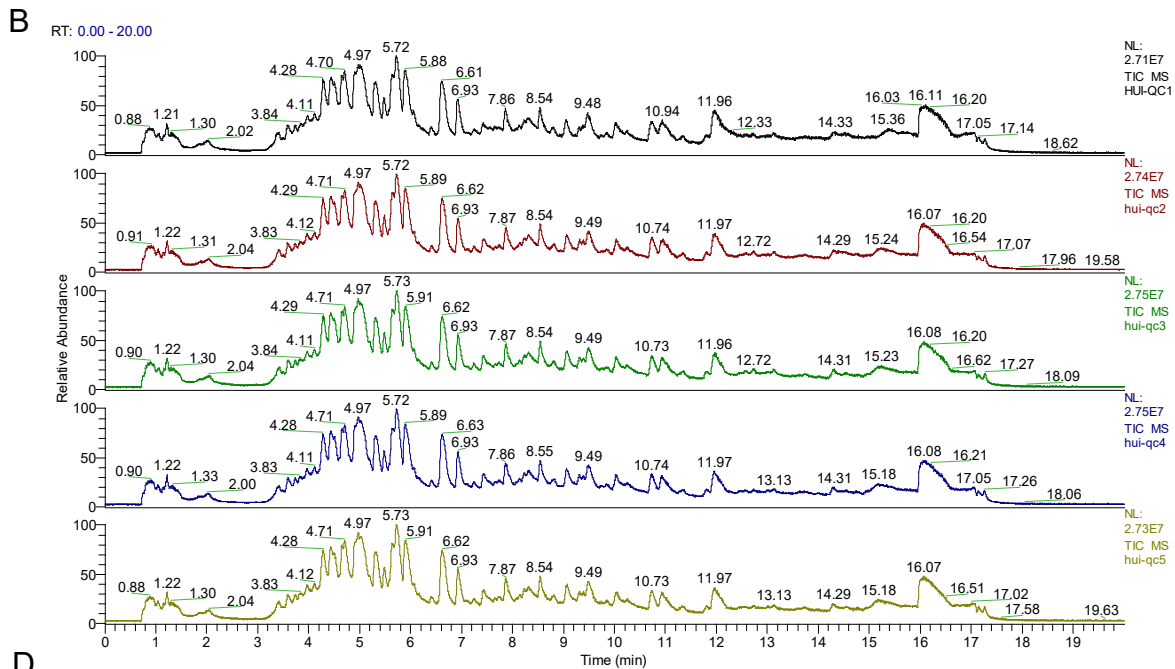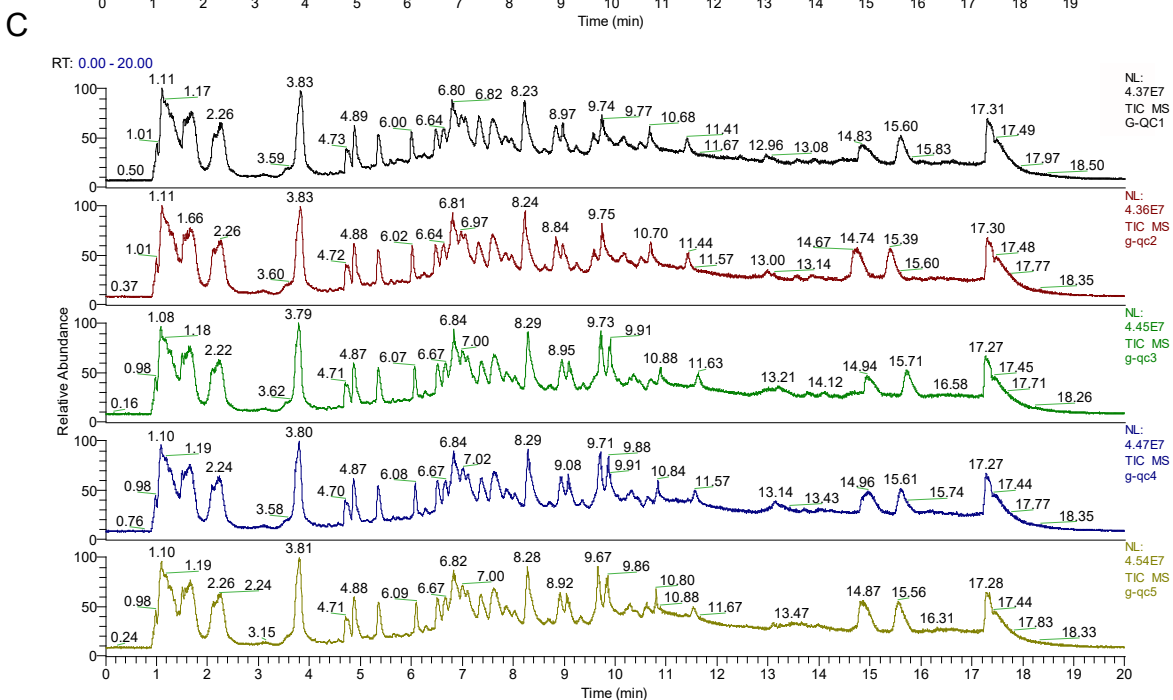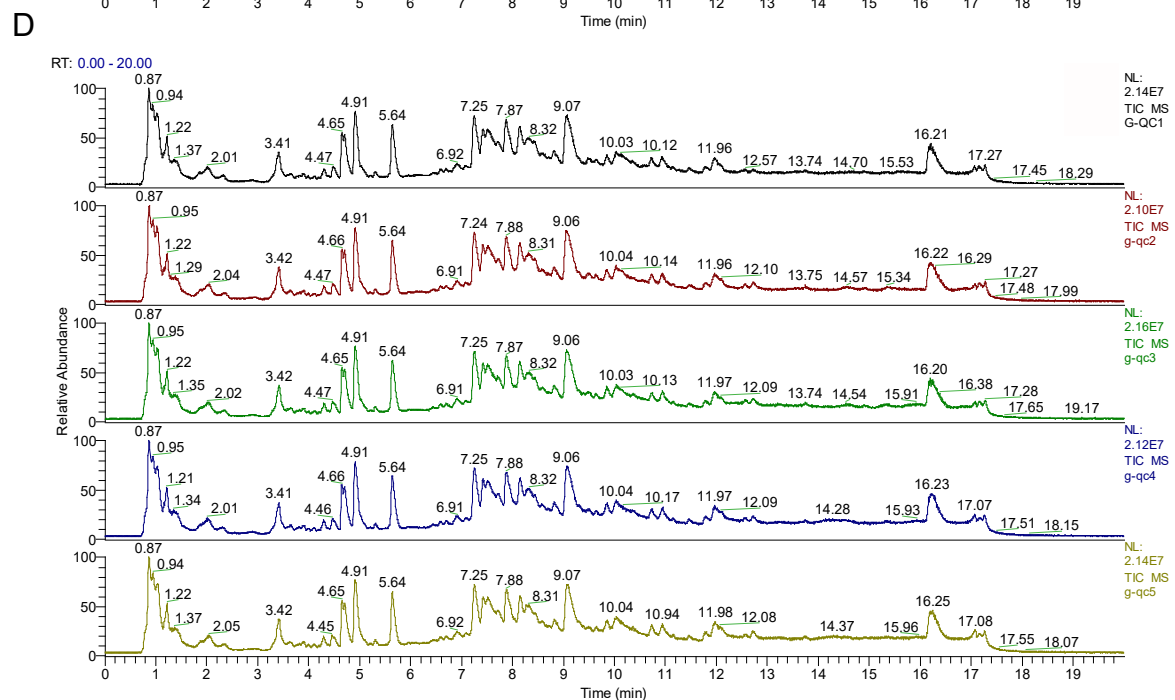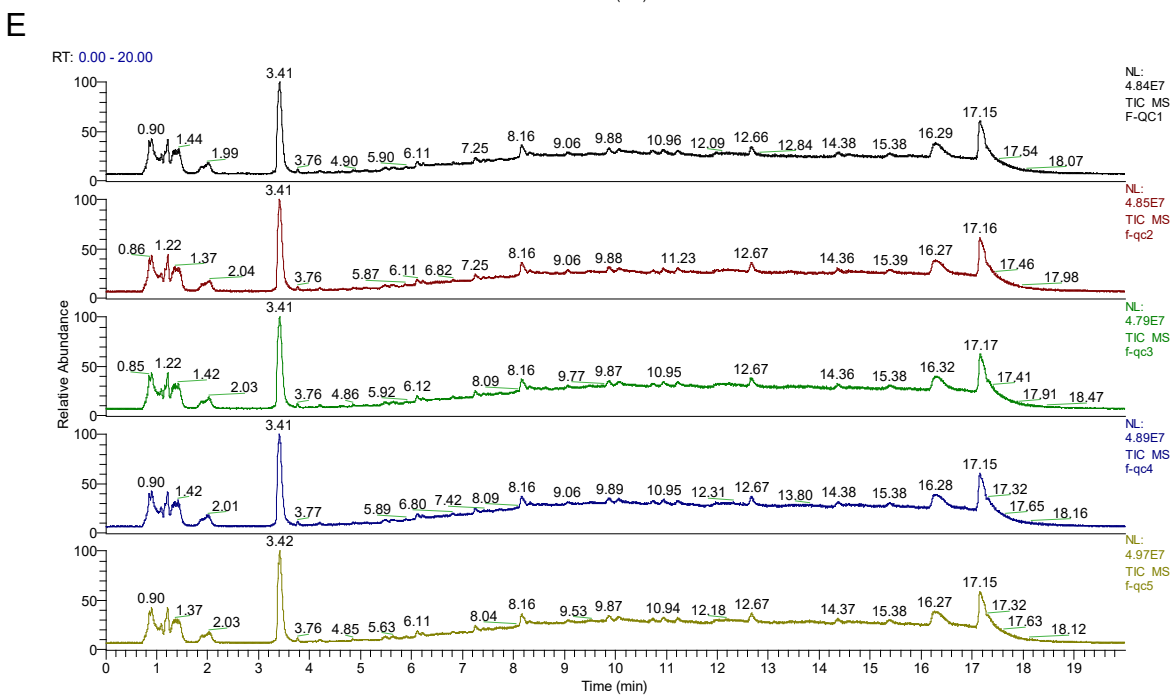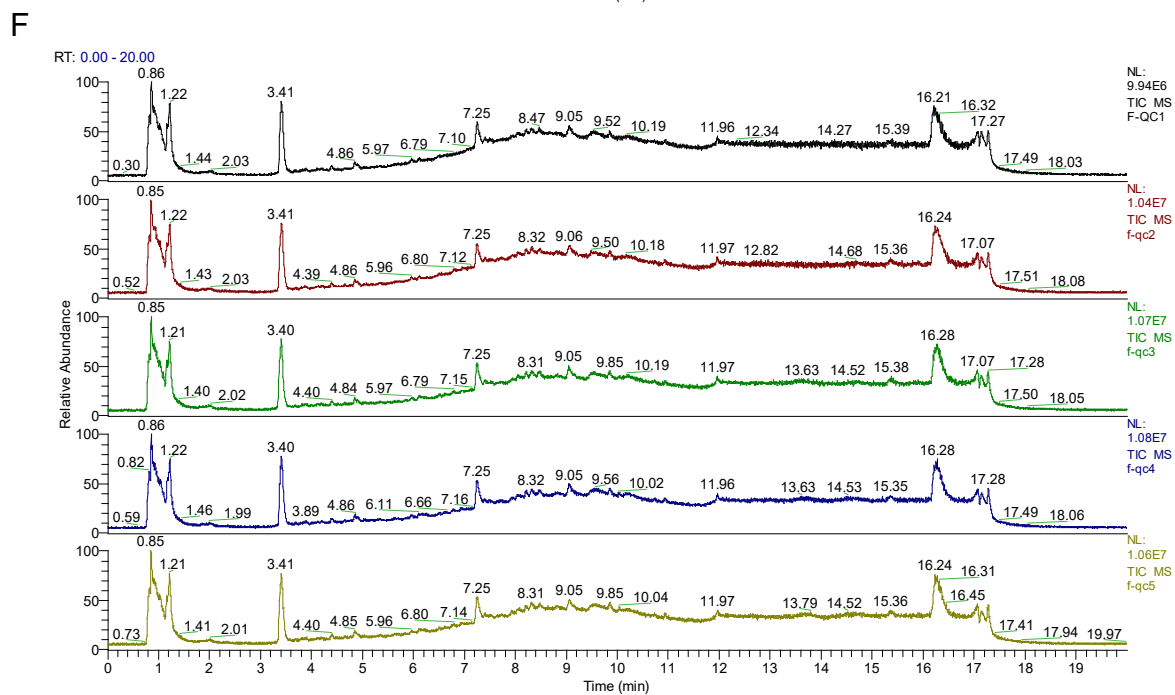

Supplement: Supplementary Figure 6 — Comparison of (A) ileal contents positive ion mode analysis, (B) ileal contents negative ion mode analysis, (C) liver positive ion mode analysis, (D) liver negative ion mode analysis, (E) abdominal aorta positive ion mode analysis, (F) abdominal aorta negative ion mode analysis in the HED and ND groups. [file Data_Sheet_6.PDF]

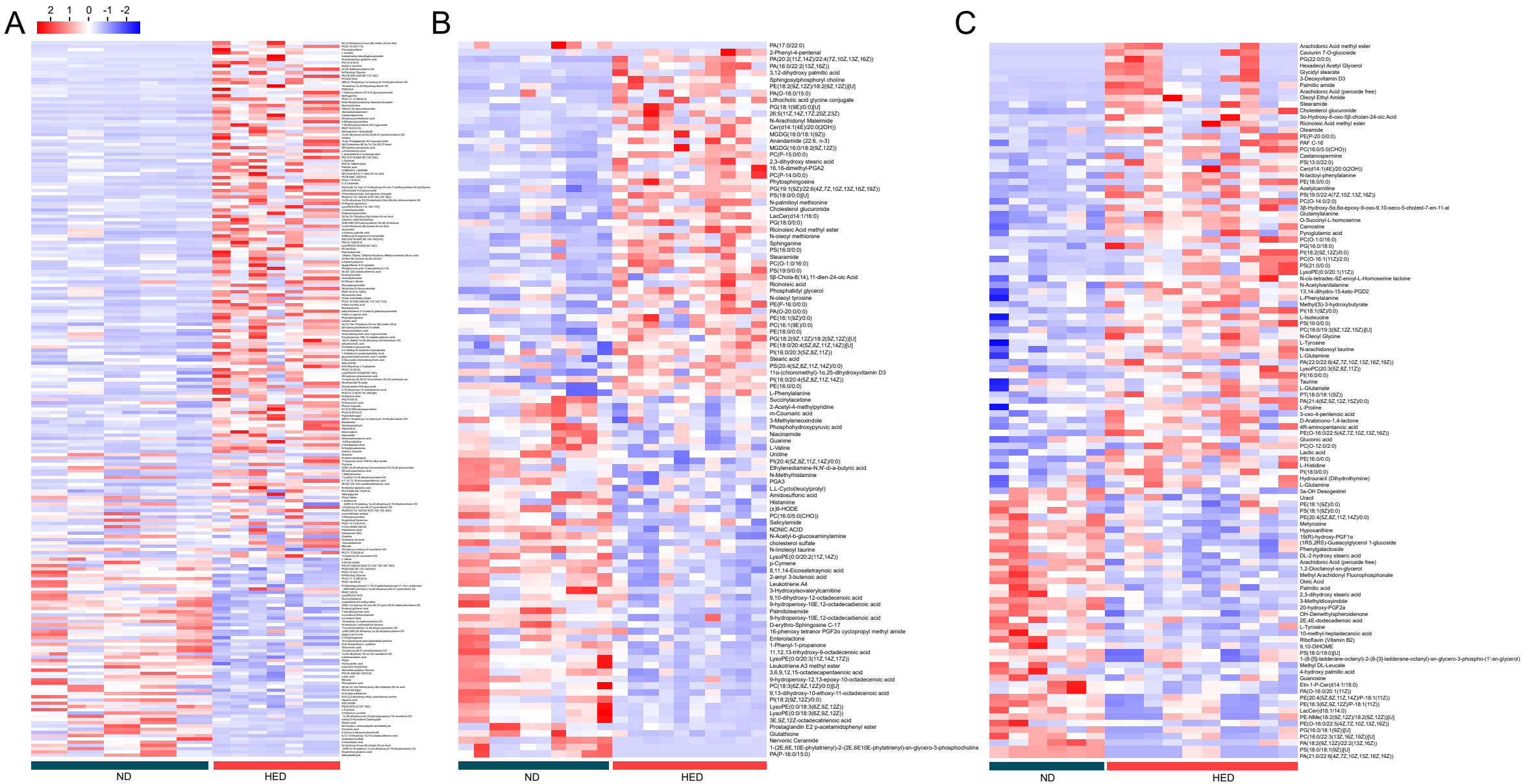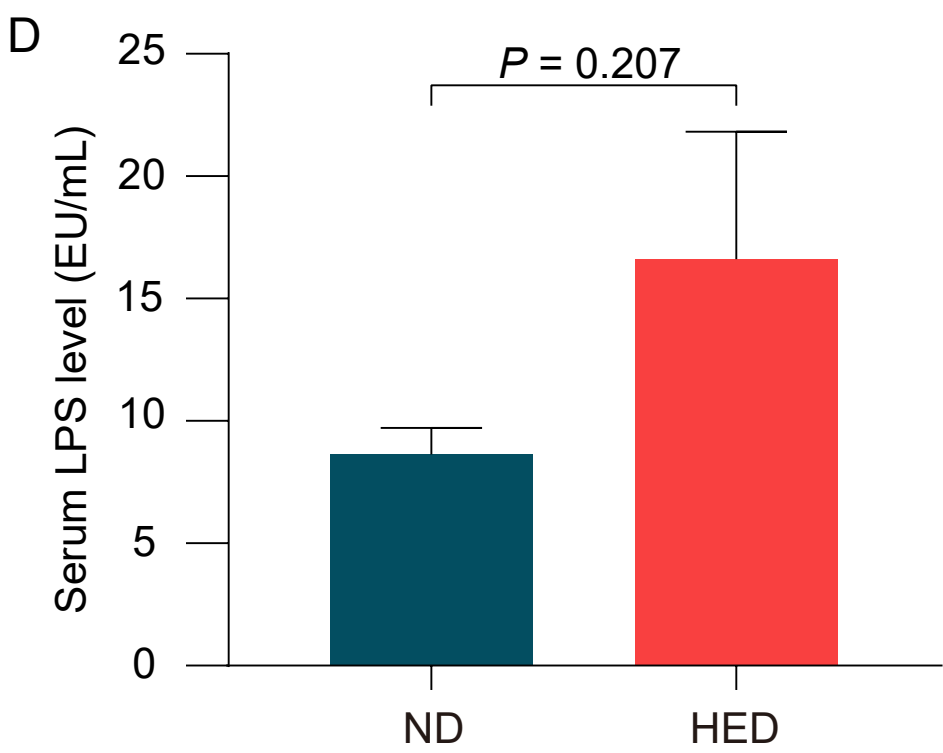

Supplement: Supplementary Figure 7 — Heatmap of untargeted ileal contents (A), liver (B), and abdominal aorta (C) tissues metabolomic analysis, showing the accumulation (abundance patterns) of the indicated annotated metabolites with significant changes (VIP > 1 and unpaired t-test P < 0.05) between the HED and ND groups (64-month experiment). (D) Quantification of serum LPS level (n = 3 in each group). Data were shown as mean ± SEM, based on student's t-test. [file Data_Sheet_7.PDF]
